# The Functional Ingredients of the Combined Extract of Mulberry Leaves and Butterfly Pea Flowers Improve Insomnia, Anxiolytic, Memory-Enhancing, and Antidepressant-like Activities in Stress-Exposed Rats

**DOI:** 10.3390/life15081308

**Published:** 2025-08-18

**Authors:** Orraya Suna, Jintanaporn Wattanathorn, Supaporn Muchimapura, Wipawee Thukham-mee, Sitthichai Iamsaard, Nongnut Uabundit

**Affiliations:** 1 Neuroscience Program, Department of Physiology and Graduate School, Faculty of Medicine, Khon Kaen University, Khon Kaen 40002, Thailand; orraya_suna@kkumail.com; 2 Department of Physiology, Faculty of Medicine, Khon Kaen University, Khon Kaen 40002, Thailand; jinwat05@gmail.com (J.W.); supmuc@kku.ac.th (S.M.); meewep@gmail.com (W.T.-m.); 3 Research Institute for High Human Performance and Health Promotion (HHP & HP), Khon Kaen University, Khon Kaen 40002, Thailand; 4 Department of Anatomy, Faculty of Medicine, Khon Kaen University, Khon Kaen 40002, Thailand; sittia@kku.ac.th

**Keywords:** anxiolytic, antidepression, insomnia, memory-enhancing, mulberry leaves, butterfly pea flowers

## Abstract

At present, a novel herbal regimen targeting anti-insomnia, anti-anxiety, cognitive performance, and anti-depression effects is required due to the limitations of the current therapy. Based on the crucial role of oxidative stress in the pathophysiology of stress-related brain disorders, it was hypothesized that the functional ingredient derived from mulberry leaves and butterfly pea flowers, which exhibits potent antioxidant activity, should protect against the disorders just mentioned. Male Wistar rats (180–200 g) were orally administered at doses of 125, 250, and 500 mg/kg BW once daily, 45 min before exposure to a 6-h immobilization stress for 14 days. Behavioral assessments, including sleep, anxiety, spatial memory, and depression, were assessed every 7 days. At the end of the study, corticosterone levels, oxidative stress markers, neurotransmitters, IL-6, BDNF, and neuron density in the prefrontal cortex and hippocampus were measured. The functional ingredients demonstrated anti-insomnia, anxiolytic, memory-enhancing, and antidepressant properties. It also increased neuron density and BDNF and activity of SOD and CAT enzymes, whereas corticosterone, GABA-T, AChE, MAO, IL-6, and MDA levels were reduced. A potential regimen targeting showed the benefits of anti-insomnia, anxiolytic, memory-enhancing, and antidepressant properties. However, further studies regarding the precise underlying mechanism and a clinical trial are essentially required.

## 1. Introduction

Currently, mental disorders, particularly insomnia, cognitive impairment, anxiety, and depression, are significantly considered global health burdens throughout the lifespan [1]. It is estimated that the loss of productivity caused by insomnia, anxiety, and depression negatively impacts the global economy by approximately 1 trillion USD per year, and it is projected that this figure will reach 16 trillion USD by 2030 [2]. According to the World Health Organization (WHO), anxiety and depression are the leading causes of disability worldwide. Additionally, reports on the cost and effectiveness of treatment indicate that preventing these mental disorders has economic value [3]. Moreover, in 2019, the global cost of dementia was estimated to be 1.3 trillion USD. The cost is projected to increase to 1.7 trillion by 2030 [4].

The physiological response to stress is characterized by increased sympathetic activity, oxidative stress, and inflammation [5]. Prolonged sympathetic activation can lead to a decrease in antioxidant enzymes, such as superoxide dismutase (SOD) and catalase [6], while promoting the release of pro-inflammatory cytokines, such as IL-6, TNF-α, and IL-1β [7]. Furthermore, repetitive stress activates the hypothalamic–pituitary–adrenal (HPA) axis, leading to increased release of stress hormones, such as cortisol [8]. Elevated cortisol levels can contribute to detrimental effects on brain plasticity, cognitive function, and neurotransmitter balance, ultimately manifesting as insomnia, mood disorders, and memory problems [9]. Studies have demonstrated that elevated oxidative stress can damage various cellular components, resulting in numerous detrimental effects, including lipid peroxidation, reduced membrane fluidity, protein degradation, and the deactivation of receptors, enzymes, and ion channels in the brain [10,11]. The existence of brain disorders can exacerbate oxidative stress and lipid peroxidation, resulting in brain damage and dysfunction [12]. Previous research suggests that antioxidants could potentially alleviate psychiatric disorders, as substances with antioxidant properties may mitigate these detrimental effects [13,14].

It is imperative to explore alternative approaches to the treatment and prevention of stress-induced mental health disorders, such as the use of medicinal plants with neuroprotective and therapeutic properties. Mulberry (*Morus alba*) leaves have high antioxidant properties, including phenolic compounds, tannins, carotenoids, and organic acids [15]. A number of studies claimed that mulberry promotes the survival and differentiation of hippocampal cells, inhibits acetylcholinesterase (AChE), and increases the formation of acetylcholine and cholinergic neurons [16,17]. Moreover, mulberry leaves have been linked to a reduction in psychiatric disorder symptoms such as anxiety and depression, as well as sedative effects [18,19]. In addition, butterfly pea (*Clitoria ternatea*) has been used to treat mental disorders [20]. The primary phytoconstituents identified in butterfly pea flowers include ternatins and anthocyanins [21]. Previous research studied the effects of a methanolic extract of butterfly pea flowers on the central nervous system of mice and rats. This extract demonstrated anti-seizure, anxiolytic, and antidepressant properties [22]. Given the potential synergistic effect of mulberry leaves and butterfly pea flowers, there has been growing interest in their combined extract as a potential treatment for neuropsychological disorders. While previous research on this combination is still unclear, this study aimed to investigate the potential benefits of the combined extract in mitigating stress-related brain disorders. We evaluated the extract’s effects on sleep induction, anxiolytic properties, memory enhancement, and antidepressant activity in stressed rats. Additionally, the underlying possible mechanisms were explored.

## 2. Materials and Methods

### 2.1. Preparation of the Functional Ingredients of the Combined Extract of Mulberry Leaves and Butterfly Pea Flowers

The mulberry leaves and butterfly pea flower extract used in this research were provided by JWF Company. The mulberry leaves of the Buriram60 variety were harvested from an agricultural plot in Khon Kaen province in September 2023. These leaves were carefully selected and labeled as KKU No. 25976. The collection site was the Khon Kaen University Herbarium. The butterfly pea flowers were identified and generously supplied by the Taxonomic Research Center at Khon Kaen University. These samples were labeled as KKU No. 25544. The combined extract was prepared in a ratio of 3:1, with mulberry leaves to butterfly pea flowers, respectively. The dietary supplement created for this study contained a concentration of polyphenolic compounds and flavonoids in the mulberry leaves and butterfly pea flower extract. These main concentrations were measured at 0.143 ± 0.003 mg/g sample of gallic acid and 0.095 ± 0.007 mg/g sample of rutin, together with 0.027 ± 0.002 mg/g sample of Peonidin-3-O-glucoside, respectively. The combined extract was stored in a dark container at 4 °C until needed. The fingerprint chromatogram of this combined extract is presented in Supplementary Materials.

### 2.2. Experimental Animals and Protocol

This study followed ethical guidelines for animal research, with all procedures involving rats approved by the Khon Kaen University Institutional Animal Care and Use Committee (IACUC-KKU-146/66). Adult male Wistar rats (6–7 weeks, 180–200 g) were obtained from the Northeast Laboratory Animal Center and housed in pathogen-free conditions at 22 ± 2 °C under a 12:12-h light/dark cycle. After a week of habituation, rats were randomly assigned to experimental groups (*n* = 6/group), as detailed in Table 1.

**Figure 1:**
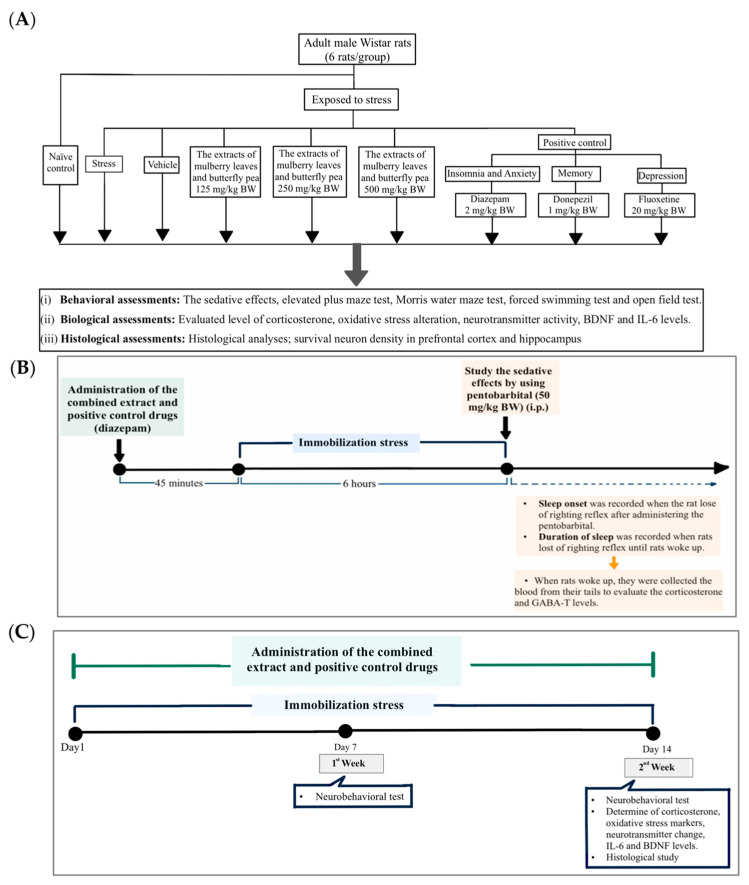
The schematic diagram explains the study protocol. (**A**) Experimental protocol of the combined extract and the determination of various parameters. (**B**) Schedule for the combined extract treatment and determination of the sleep assessment on the first day of this study. (**C**) Schedule for the combined extract treatment and determination of the anxiety, memory, and depression like-activities.

All rats received their designated treatments 45 min before being exposed to 6 h of immobilization stress daily for two weeks (except for the naïve control group). Immobilization was achieved using a restricted stainless-steel cage, allowing no movement. Stress sessions occurred from 8 AM to 2 PM. All substances were administered orally once daily for two weeks using an oral gavage needle. Following this, behavioral assessments were conducted on rats for hypnotic, anxiety, spatial memory, and depression responses, together with the open field test. These assessments were conducted on days 1, 7, and 14 (except for the sedative effect test, which was only performed on the first day). At the end of the study, rats were evaluated for oxidative stress markers, including malondialdehyde (MDA) levels and the activities of catalase (CAT) and superoxide dismutase (SOD). Neurotransmitter enzyme changes were examined by measuring acetylcholinesterase (AChE) and monoamine oxidase (MAO) levels in the prefrontal cortex and hippocampus. Additionally, corticosterone and GABA-transaminase (GABA-T) levels were investigated in both serum and brain samples. Finally, neuron density, inflammatory markers (IL-6), and brain-derived neurotrophic factor (BDNF) were also evaluated in brain samples, as detailed in Figure 1A–C.

### 2.3. Behavioral Assessments

#### 2.3.1. Sleep Assessment

Sleep assessment was conducted by administering pentobarbital intraperitoneally at a dose of 50 mg/kg after immobilization stress. Sleep onset was immediately determined as the time after injection until its loss of the righting reflex. Sleep duration, the time from loss of righting reflex to recovery, was recorded [23].

#### 2.3.2. Elevated Plus Maze Test (EPM)

Rats were evaluated for anxiolytic effects using the elevated plus maze, which measures anxiety based on rodents’ aversion to open, elevated areas. The maze had two open and two enclosed arms (each 50 × 10 cm; enclosed arms had 40 cm high walls) elevated 50 cm above the floor. Each rat started in the center facing an open arm. Behavior was video recorded for 5 min, and the maze was cleaned between tests. Entries and time spent in open arms were recorded on days 7 and 14 to assess anxiolytic activity [24].

#### 2.3.3. Morris Water Maze Test (MWM)

In this procedure, rats were trained to locate a hidden platform submerged within a circular pool, relying on surrounding environmental cues for navigation. Training consisted of two trials per day over four consecutive days. To evaluate learning and memory, the assessment included an acquisition phase measuring escape latency, followed by a probe trial 24 h later, during which the platform was removed and retention time was recorded. These assessments were conducted both before and after the intervention, specifically on days 7 and 14 [25].

#### 2.3.4. Forced Swimming Test (FST)

The antidepressant-like effects of the combined extract were measured using the forced swim test. In this method, rats are placed in a cylinder of water at 25 °C, unable to touch the bottom or escape, for six minutes. Their periods of immobility and active movement are recorded. The test is performed on days 7 and 14 after treatment [26].

#### 2.3.5. Open Field Test (OFT)

Locomotion and exploration were measured using the open field test. Each rat was placed in a square arena marked with a grid, and behaviors such as rearing, grooming, line crossings, and entries into the center square were counted for 5 min. The arena was cleaned between trials. Diazepam was used as a positive control [27].

### 2.4. Biochemical Assessments

#### 2.4.1. Measurement of Oxidative Stress Markers

After sacrifice, the brains were carefully extracted and homogenized in ice-cold 0.1 M phosphate-buffered saline at a buffer-to-tissue volume ratio of 50:1. The resulting homogenate was subjected to centrifugation at 3000× *g* for 15 min at 4 °C. The resulting supernatant was used for further analytical procedures. This study measured malondialdehyde (MDA), a marker of lipid peroxidation, as well as key endogenous antioxidant enzymes, superoxide dismutase (SOD) and catalase (CAT), as indicators of oxidative stress in brain tissue, as described by Thongwong and coworkers [28].

#### 2.4.2. Measurement of Neurotransmitter Enzyme Changes

In this study, acetylcholinesterase (AChE) activity was measured as an indirect marker of cholinergic system function, following the protocol established by Wattanathorn and coworkers [29]. A 20 µL sample of brain homogenate was combined with a reaction mixture containing 0.1 mM sodium phosphate (200 µL) and 0.2 M of DTNB (10 µL). This mixture was incubated at room temperature for 5 min. Subsequently, 10 µL of 15 mM acetylcholine trichloride was added, followed by an additional 3-min incubation. The absorbance was then measured at 412 nm using a microplate reader.

Monoamine oxidase (MAO) activity was determined by monitoring the formation of a red quinonimine dye, produced when 4-aminoantipyrine is oxidized and subsequently reacts with vanillic acid. To perform the assay, 50 µL of the sample was mixed in a 96-well microplate with a chromogenic solution dissolved in 0.2 M potassium phosphate buffer (pH 7.6), along with 50 µL of a chromium solution and 200 µL of 500 µM p-tyramine (Sigma-Aldrich, Saint Louis, MO, USA). After incubating for 30 min at room temperature, absorbance was measured at 490 nm using a microplate reader. The results were expressed as µmol of product per mg of protein [30].

GABA-T activity was assessed using an ELISA kit from My BioSource Inc. (San Diego, CA, USA) according to the manufacturer’s protocol. Briefly, 40 µL of each sample was combined with 10 µL of biotinylated antibody and 50 µL of streptavidin-HRP in a microplate well, followed by incubation at 37 °C for 1 h. After washing the wells five times with 300 µL of wash buffer, 50 µL each of solutions A and B was added, and the plate was incubated at 37 °C for an additional 10 min. The reaction was stopped by adding 50 µL of stop solution, and absorbance was read at 450 nm.

#### 2.4.3. Measurement of Corticosterone

Corticosterone levels in serum and brain samples were determined using an ELISA kit (My BioSource Inc., San Diego, CA, USA) according to the manufacturer’s instructions.

#### 2.4.4. Assessment of Inflammatory Marker and Neuronal Marker

Inflammatory cytokines, interleukin-6 (IL-6), were measured using ELISA kits (ABclonal Inc., Wuhan, China). All assessments followed the manufacturer’s protocols. In brief, a 100 µL sample aliquot was mixed with 100 µL of EIA buffer and 100 µL of standard in a 96-well microplate coated with biotinylated primary antibodies. After a 2-h incubation at 37 °C, the plate was washed three times with wash buffer. Streptavidin-horseradish peroxidase conjugate solution (100 µL) was added to each well and incubated for 30 min at 37 °C, followed by another wash. Substrate solution (100 µL) was added to each well and incubated for 20 min at 37 °C. Finally, 50 µL of stop solution was added, and absorbance was measured at 450 nm.

Neuronal marker, Brain-Derived Neurotrophic Factor (BDNF), was measured using ELISA kits (My BioSource, Inc., San Diego, CA, USA). All assessments followed the manufacturer’s protocols.

### 2.5. Histological Procedure and Nissl Staining

Brains were perfused with a 4% paraformaldehyde (Sigma-Aldrich, USA) solution in 0.1 M phosphate buffer (pH 7.4). After post-fixation overnight at 4 °C, brains were infiltrated with 30% sucrose for 72 h. Frozen brain sections (10 µm thick) were then cut using a cryostat. Sections were mounted on gelatin-coated slides and stained with cresyl violet (Sigma-Aldrich, USA). Dehydration was performed using graded alcohol baths of 70%, 95%, and 100%, with two changes at each concentration, followed by clearing in xylene and mounting with DPX. Based on stereotaxic coordinates, three representative sections were selected from the rat brain atlas for the medial prefrontal cortex (mPFC) and hippocampus. Neuron density was evaluated for layer 4/5 pyramidal neurons in the mPFC (anteroposterior 2.5–4.5 mm, mediolateral 0.2–1.0 mm) and in the hippocampus (3.70 mm posterior to bregma, ± 2.2 mm mediolateral, and 3.4 mm dorsoventral), using light microscopy at 40× magnification [31].

### 2.6. Statistical Analysis

Data are presented as mean ± standard error of the mean (SEM). Statistical comparisons between groups were conducted using one-way analysis of variance (ANOVA), followed by Tukey’s post-hoc test to identify specific group differences. Statistical significance was determined by *p*-values less than 0.05. All analyses were conducted using SPSS software, version 28.0.1.0.

## 3. Results

### 3.1. Behavioral Assessment

#### 3.1.1. The Sedative Effect

**Figure 2:**
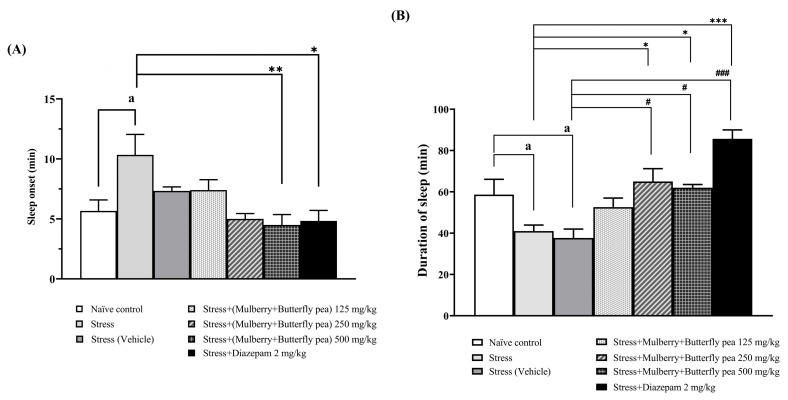
The effect of the combined extract of mulberry leaves and butterfly pea flowers on the pentobarbital-induced sedative effect. (**A**) Onset of sleep, (**B**) Duration of sleep. Data are presented as mean ± SEM (*n* = 6/group). ^a^ *p* < 0.05 when compared with the naïve control group. *^,^ **^,^ *** *p* < 0.05, <0.01 and <0.001 when compared with stress group. ^#, ###^ *p* < 0.05, <0.001 when compared with stress + vehicle group.

The sedative effects of a combined extract of mulberry leaves and butterfly pea flowers on stress-induced rats were evaluated (Figure 2A,B). Pentobarbital-induced sleep was used to determine the sedative effect. Rats in the stress group showed increased latency to sleep onset and decreased duration of sleep. Rats administered the combined extract at the highest dose showed a significant decrease in sleep onset latency. Additionally, rats given the extract at doses of 250 or 500 mg/kg BW demonstrated a significantly increased duration of sleep. Moreover, rats treated with diazepam exhibited a significantly decreased sleep onset latency and increased duration of sleep.

#### 3.1.2. The Anxiolytic Effect

**Figure 3:**
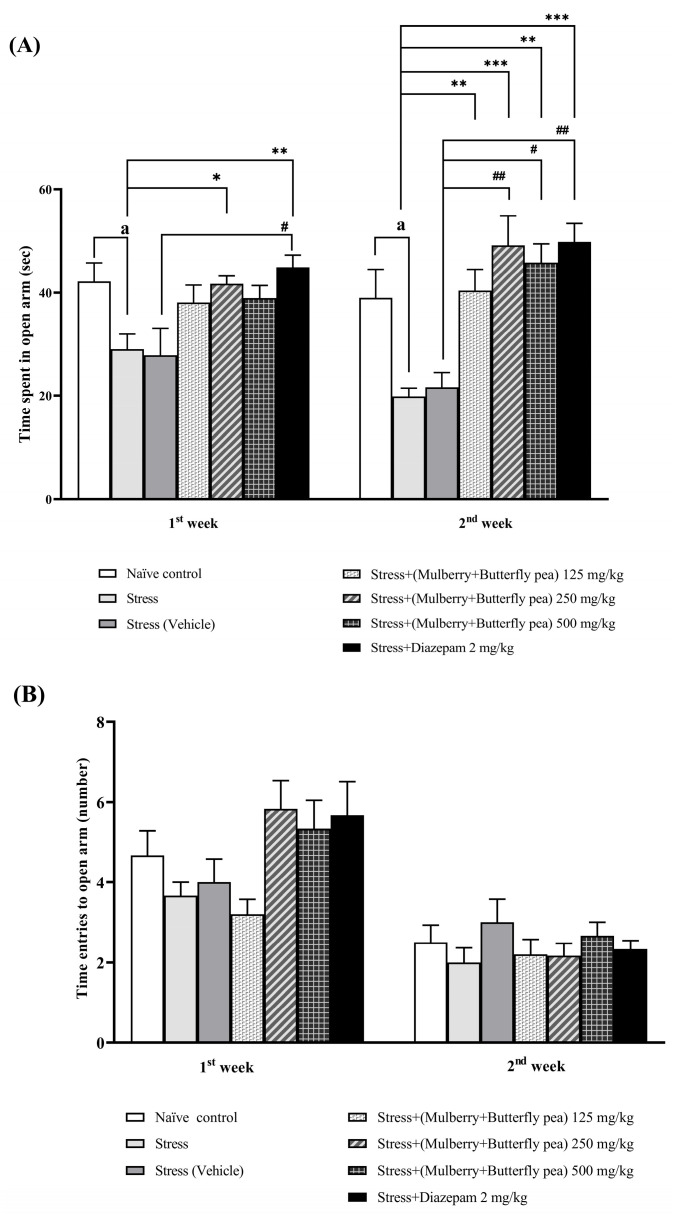
The effect of the combined extract of mulberry leaves and butterfly pea flowers on the anxiolytic effect. (**A**) Time spent in open arms. (**B**) Time entries to open arm. Data are presented as mean ± SEM (*n* = 6/group). ^a^ *p* < 0.05 when compared with the naïve control group. *^,^ **^,^ *** *p* < 0.05, <0.01 and <0.001 when compared with stress group. ^#, ##^ *p* < 0.05 and < 0.01 when compared with stress + vehicle group.

The anxiolytic effects of the combined mulberry leaves and butterfly pea flower extract were evaluated using the elevated plus maze (EPM) test in rats. The time spent in the open arms and the number of open arm entries were recorded to assess anxiety levels. The combined extract significantly increased the time spent in the open arms of the maze, particularly at a dose of 250 mg/kg BW in the first week (Figure 3A). Diazepam-treated rats also showed increased open arm time. Additionally, all doses of the combined extract (125, 250, and 500 mg/kg BW) significantly increased open arm time in the second week. However, there were no significant changes in the number of open arm entries for diazepam-treated rats or those receiving the combined extract (Figure 3B).

#### 3.1.3. The Memory Enhancing Effect

**Figure 4:**
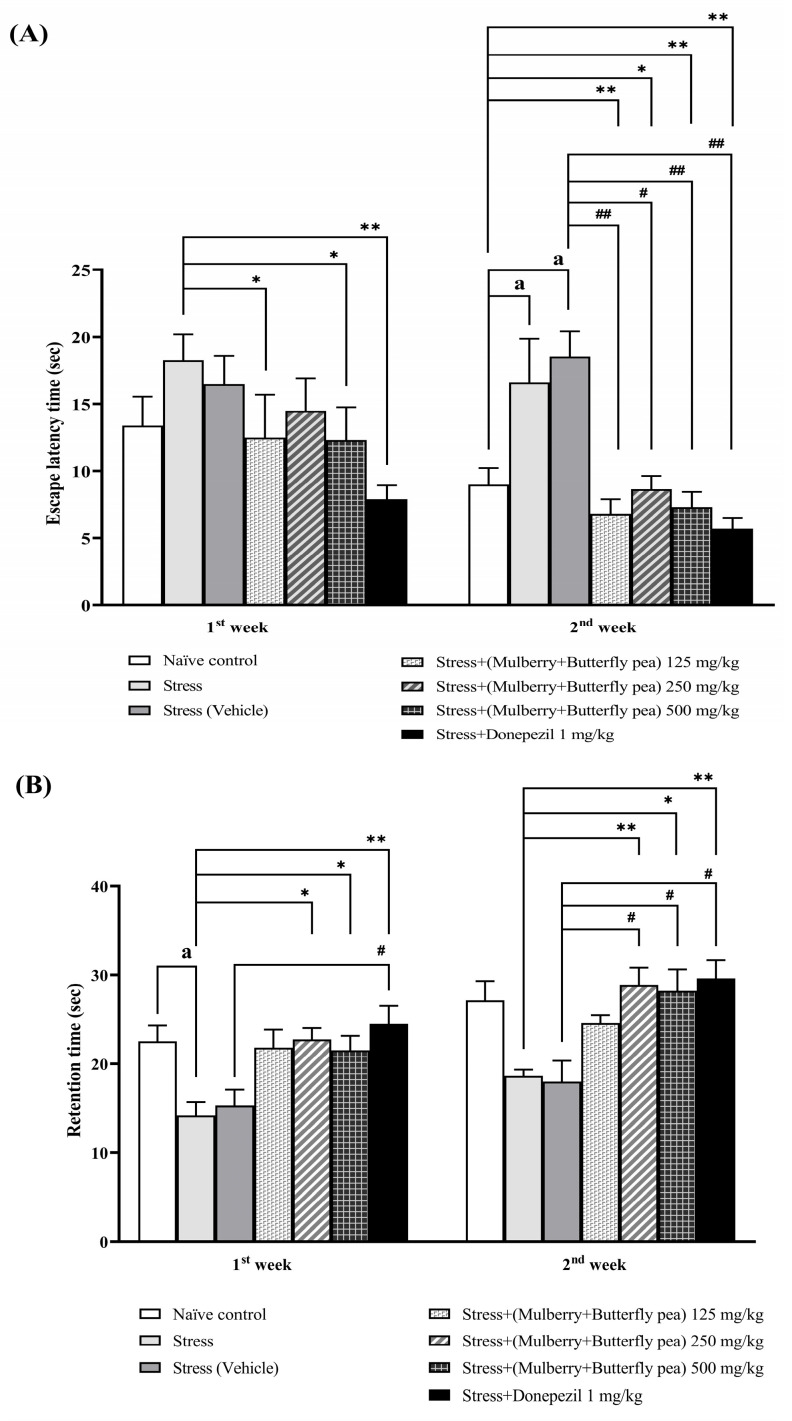
The effect of the combined extract of mulberry leaves and butterfly pea flowers on the memory-enhancing effect. (**A**) Escape latency time. (**B**) Retention time. Data are presented as mean ± SEM (*n* = 6/group). ^a^ *p* < 0.05 when compared with the naïve control group. *^,^ ** *p* < 0.05 and <0.01 when compared with stress group. ^#, ##^ *p* < 0.05 and <0.01 when compared with stress + vehicle group.

The effects of the combined mulberry leaves and butterfly pea flower extract on spatial memory were evaluated using the Morris water maze test. Figure 4A shows that doses of 125 and 500 mg/kg BW significantly reduced escape latency time in the first week. By the second week, all doses significantly decreased the escape latency time. Additionally, Figure 4B shows that doses of 250 and 500 mg/kg BW significantly increased retention time in both the first and second weeks. The donepezil-treated group showed decreased escape latency and increased retention time throughout the 14-day period.

#### 3.1.4. The Antidepressant Effect

**Figure 5:**
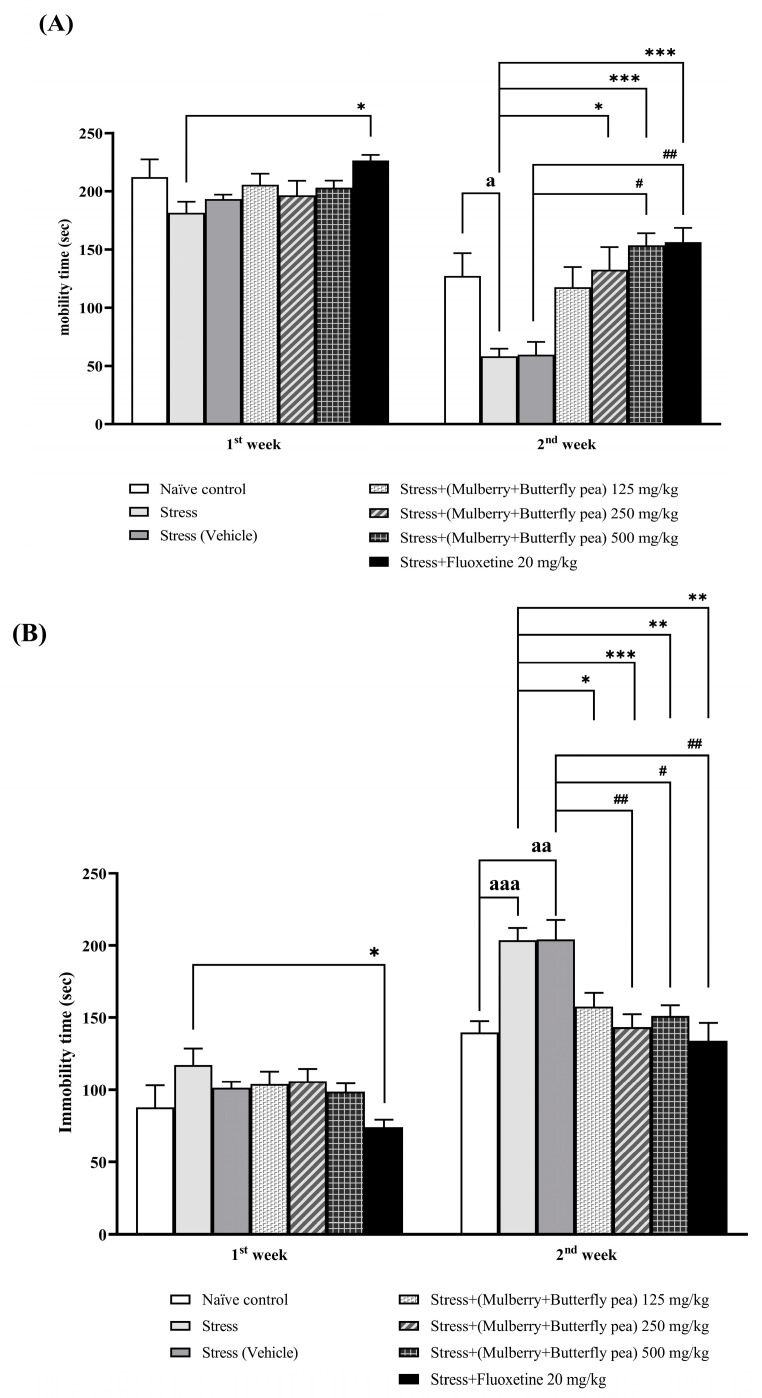
The effect of the combined extract of mulberry leaves and butterfly pea flowers on the antidepressant effect. (**A**) Mobility time, (**B**) Immobility time. Data are presented as mean ± SEM (*n* = 6/group). ^a, aa, aaa^ *p* < 0.05 when compared with naïve control group. *^,^ **^,^ *** *p* < 0.05, <0.01 and <0.001 when compared with stress group. ^#, ##^ *p* < 0.05 and <0.01 when compared with the stress (vehicle) group.

The antidepressant effects of the combined mulberry leaves and butterfly pea flower extract were evaluated using the forced swim test, a well-established method for assessing antidepressant activity. Figure 5A presents the effects of various doses of the combined extract on mobility time in the forced swim test. In the first week, rats receiving different doses of the combined extract did not exhibit significant changes in mobility or immobility time. However, by the second week, all doses significantly increased mobility time and decreased immobility time (Figure 5B).

#### 3.1.5. Locomotor Activity

**Figure 6:**
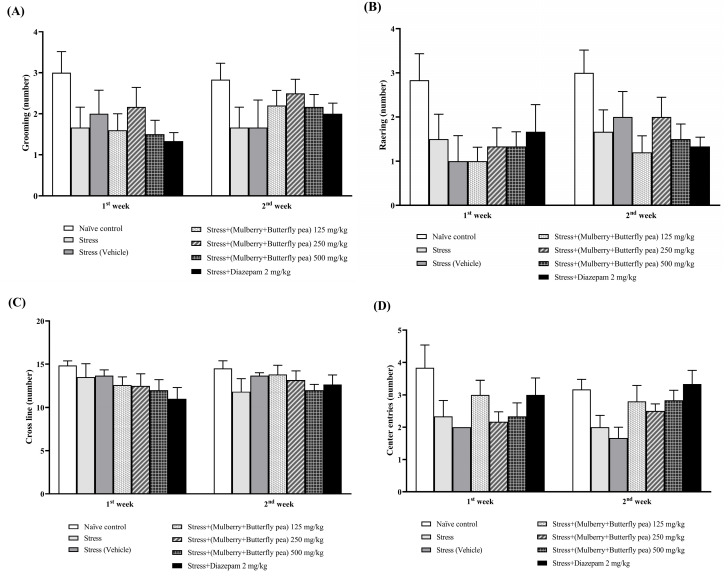
The effect of the combined extract of mulberry leaves and butterfly pea flowers on the Antidepressant Effect. (**A**) Grooming, (**B**) Rearing, (**C**) Cross line, and (**D**) Center entries. Data are presented as mean ± SEM (*n* = 6/group).

The effect of various doses of the combined extract of mulberry leaves and butterfly pea flowers on grooming, rearing, cross line, and center entries behaviors is shown in Figure 6A–D. The present results showed that no significant changes in grooming, rearing, cross line, and center were observed in the combined extract-treated group. Therefore, our results suggested that the combined extract exerted no effect on locomotor activity.

### 3.2. Changes in Oxidative Stress Markers

The alterations of malondialdehyde (MDA) level in the prefrontal cortex and hippocampus were also explored, and the results are shown in Table 2 and Table 3. In the prefrontal cortex, the results demonstrated that the combined mulberry leaves and butterfly pea flowers at a dose of 125 mg/kg BW had no significant change in MDA level. However, this change was decreased by medium and highest doses of the combined extract group. Interestingly, in the hippocampus, the results demonstrated that the combined extract of all doses significantly decreased on MDA level. In addition, the endogenous scavenging activities, including superoxide dismutase (SOD) and catalase (CAT), were observed in the prefrontal cortex and hippocampus. The results showed that the stress group decreased SOD activity in both areas. Moreover, in the prefrontal cortex, a significant increase in SOD activity was found at doses of 250 and 500 mg/kg BW of the combined extract. In the hippocampus, the combined extract of mulberry leaves and butterfly pea flowers at all doses significantly increased SOD activity. However, the results demonstrated that the combined extract of mulberry leaves and butterfly pea flowers at doses of 125 and 250 mg/kg BW did not result in a significant increase in CAT activity. Fortunately, the highest dose of the combined extract significantly increased CAT activity.

### 3.3. Neurotransmitters Enzyme Changes

**Figure 7:**
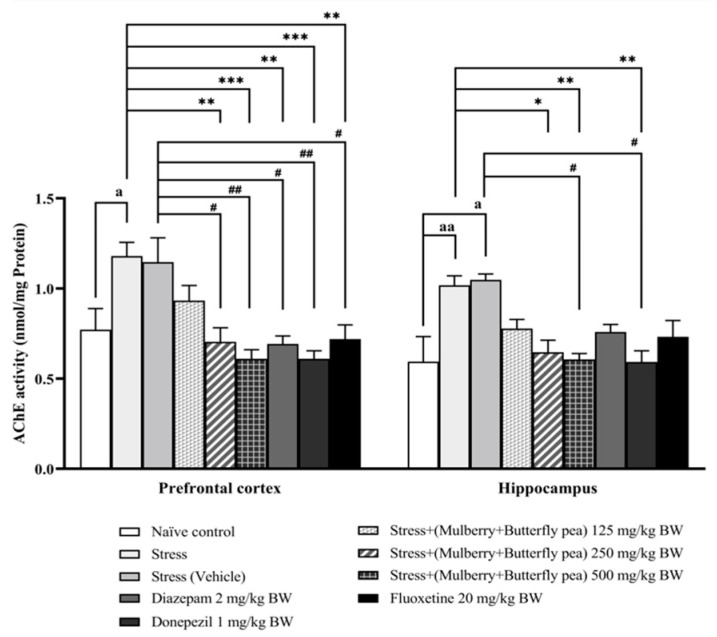
Acetylcholinesterase (AChE) activity in the prefrontal cortex and hippocampus of various treatment groups. Data are presented as mean ± SEM (*n* = 6/group). ^a, aa^ *p* < 0.05 when compared with naïve control group. *^,^ **^,^ *** *p* < 0.05, <0.01 and <0.001 when compared with stress group. ^#, ##^ *p* < 0.05 and <0.01 when compared with the stress (vehicle) group.

**Figure 8:**
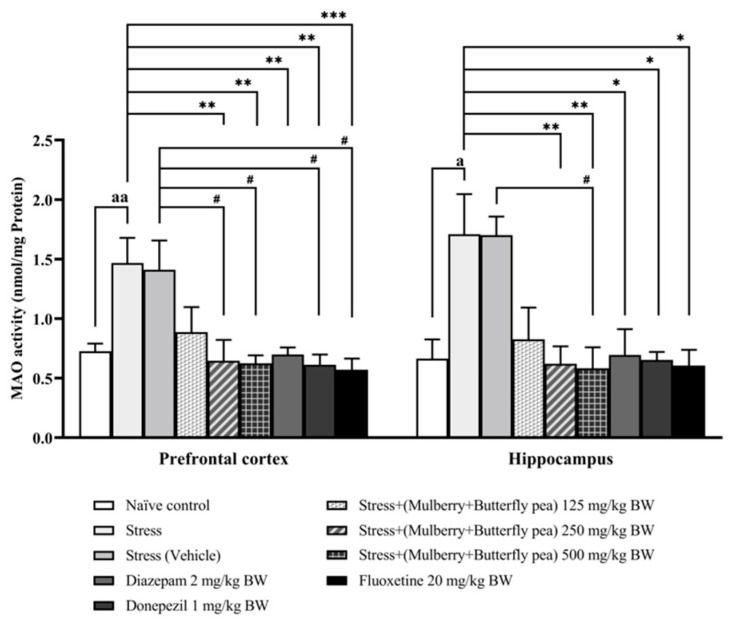
Monoamine oxidase (MAO) activity in the prefrontal cortex and hippocampus of various treatment groups. Data are presented as mean ± SEM (*n* = 6/group). ^a, aa^ *p* < 0.05 and <0.01 when compared with naïve control group. *^,^ **^,^ *** *p* < 0.05, < 0.01, and <0.001 when compared with stress group. ^#^ *p* < 0.05 when compared with stress + vehicle group.

**Figure 9:**
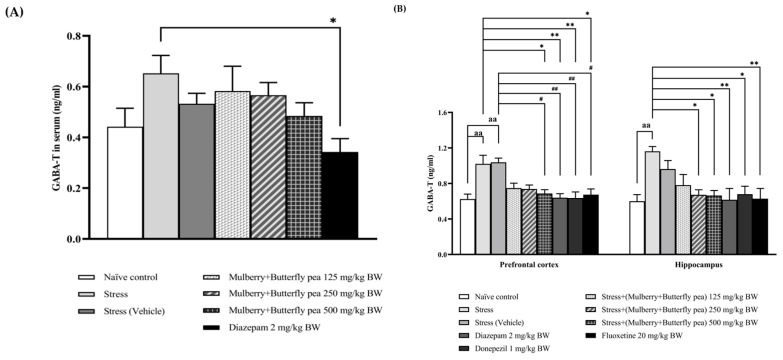
GABA-transaminase (GABA-T) activity. (**A**) GABA-T in serum. (**B**) GABA-T in the prefrontal cortex and hippocampus of various treatment groups. Data are presented as mean ± SEM (*n* = 6/group). ^aa^ *p* < 0.01 when compared with naïve control group. *^,^ ** *p* < 0.05 and <0.01 when compared with stress group. ^#, ##^ *p* < 0.05 and <0.01 when compared with stress + vehicle group.

Changes in neurotransmitter levels have been associated with alterations in memory and mood. The activities of key enzymes involved in the metabolism of these neurotransmitters, acetylcholinesterase (AChE) for acetylcholine, monoamine oxidase (MAO) for monoamines, and GABA-transaminase (GABA-T) for gamma-aminobutyric acid (GABA), were measured as indirect indicators of their turnover rates. The corresponding results are presented in Figure 7, Figure 8 and Figure 9.

The alterations of AChE activity in the prefrontal cortex and hippocampus are also explored, and the results are shown in Figure 7. In the prefrontal cortex, the stress group significantly increased AChE activity, whereas the stress + vehicle group failed to show significantly increased AChE activity. However, this change was mitigated by medium and highest doses (250 and 500 mg/kg BW) of the combined extract and donepezil group. In the hippocampus, the stress group and the stress + vehicle group significantly increased AChE activity. The results demonstrated that the combined extract of mulberry leaves and butterfly pea flowers, only at the highest dose (500 mg/kg BW), and donepezil significantly decreased the activity of acetylcholinesterase.

Figure 8 presents the changes in MAO activity within the prefrontal cortex and hippocampus. In the prefrontal cortex, the stress group demonstrated a significant increase in MAO activity. The combined extract of mulberry leaves and butterfly pea flowers at the low dose (125 mg/kg BW) did not produce significant changes in MAO activity. However, the medium and high doses of the combined extract significantly reduced this stress-induced elevation in MAO activity. In the hippocampus, the stress group showed significantly increased MAO activity. The combined extract at medium and high doses (250 and 500 mg/kg BW) significantly reduced MAO activity compared to the stress group. The positive control drugs—diazepam, donepezil, and fluoxetine—also significantly decreased MAO activity in both brain regions.

The alterations of GABA-transaminase (GABA-T) activity in serum were also explored, and the results are presented in Figure 9A. The effect of the combined extract of mulberry leaves and butterfly pea flowers on pentobarbital-induced sedative activity was assessed. The stress group and stress + vehicle group did not show a significant decrease in GABA-T activity. Furthermore, the results demonstrated that all doses of the combined extract of mulberry leaves and butterfly pea flowers (125, 250, and 500 mg/kg BW) produced no significant changes in GABA-T activity.

The alterations of GABA-transaminase (GABA-T) activity in the prefrontal cortex and hippocampus were measured, and the results are presented in Figure 9B. In the prefrontal cortex, the stress group and stress + vehicle group showed a significant increase in GABA-T activity. The combined extract of mulberry leaves and butterfly pea flowers at doses of 125 and 250 mg/kg BW did not produce a significant change in GABA-T activity. However, this increase was mitigated by the highest dose (500 mg/kg BW) of the combined extract, as well as by diazepam, donepezil, and fluoxetine. In the hippocampus, the stress group also showed a significant increase in GABA-T activity. The combined extract at medium and highest doses (250 and 500 mg/kg BW) significantly decreased GABA-T activity. In addition, the positive control drugs—diazepam, donepezil, and fluoxetine—also significantly decreased GABA-T activity.

### 3.4. Hormonal Essay

**Figure 10:**
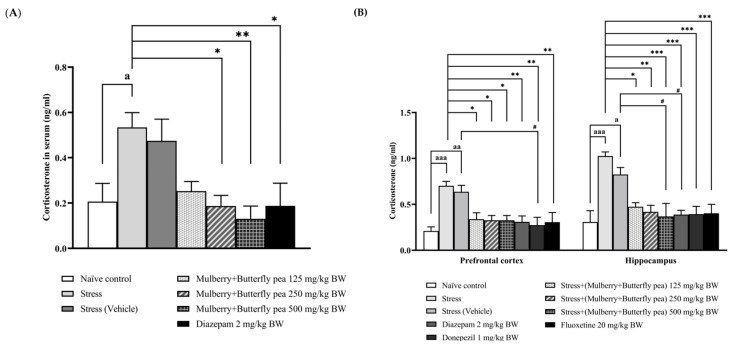
Levels of (**A**) corticosterone in serum and (**B**) corticosterone in the prefrontal cortex and hippocampus of various treatment groups. Data are presented as mean ± SEM (*n* = 6/group). ^a, aa, aaa^ *p* < 0.05, <0.01, and <0.001 when compared with naïve control group. *^,^ **^,^ *** *p* < 0.05, <0.01 and <0.001 when compared with stress group. ^#^ *p* < 0.05 when compared with stress + vehicle group.

Corticosterone levels in serum and brain samples were measured, and the results are presented in Figure 10A,B. In serum, the combined mulberry leaves and butterfly pea flowers extract did not significantly alter corticosterone levels in the stress or vehicle groups. However, doses of 250 and 500 mg/kg BW significantly decreased corticosterone levels compared to the stress group.

The stress and vehicle groups exhibited significantly increased corticosterone levels in the prefrontal cortex. In contrast, the combined extract at all doses significantly decreased corticosterone levels in the prefrontal cortex. In addition, diazepam, donepezil, and fluoxetine also decreased corticosterone levels in the prefrontal cortex. In the hippocampus, the stress group showed significantly increased corticosterone levels. Conversely, the combined extract at all doses significantly decreased corticosterone levels.

### 3.5. Assessment of Inflammatory Marker and Neuronal Biomarker

**Figure 11:**
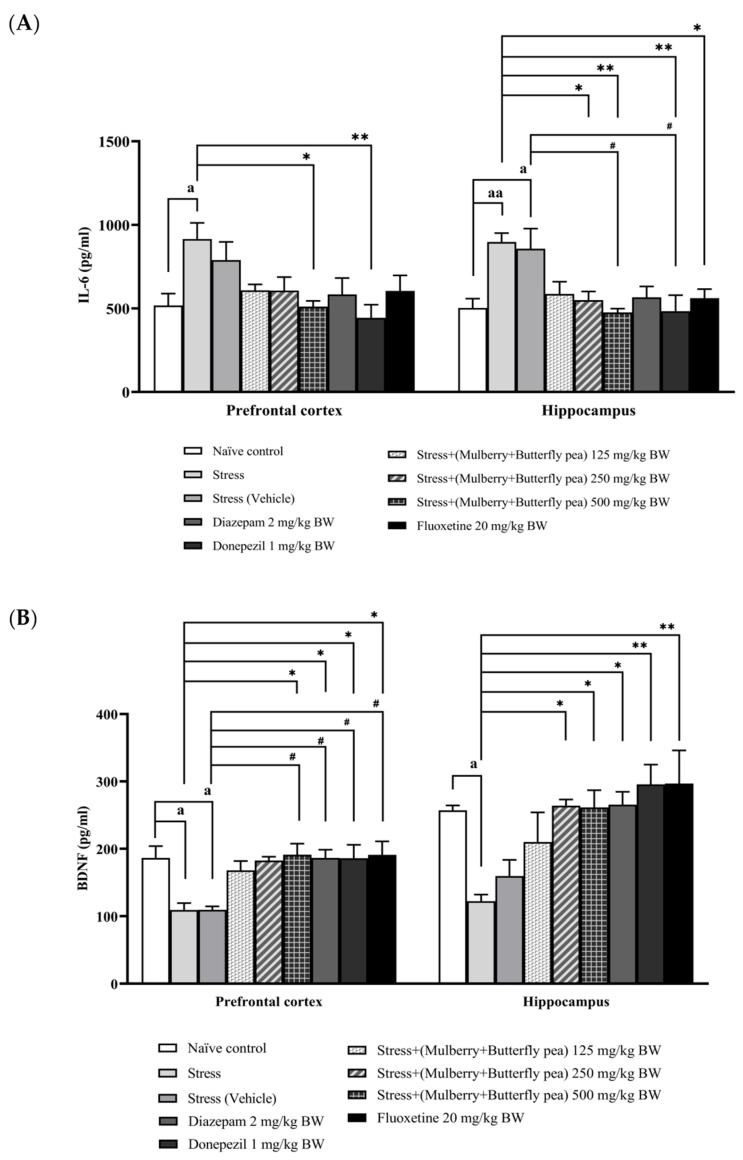
Levels of (**A**) IL-6 and (**B**) BDNF in the prefrontal cortex and hippocampus of various treatment groups. Data are presented as mean ± SEM (*n* = 6/group). ^a, aa^ *p* < 0.05 and <0.01 when compared with the naïve control group. *^,^ ** *p* < 0.05 and <0.01 when compared with stress group. ^#^ *p* < 0.05 when compared with stress + vehicle group.

Because neuroinflammation contributes to cognitive impairment, we evaluated the effects of the combined mulberry leaves and butterfly pea flower extract on interleukin-6 (IL-6) levels in the prefrontal cortex and hippocampus (Figure 11A). Exposure to stress resulted in a significant elevation of IL-6 levels in both brain regions. Treatment with low and medium doses of the combined extract did not result in a significant reduction in IL-6 concentrations in either the prefrontal cortex or hippocampus. Fortunately, administration of the highest dose of the combined extract led to a significant decrease in IL-6 levels in both regions. Among the positive control treatments, donepezil and fluoxetine significantly lowered IL-6 in the hippocampus, whereas diazepam did not produce a significant reduction in IL-6 in either the prefrontal cortex or the hippocampus.

In this experiment, we also investigated the effect of the combined extract of mulberry leaves and butterfly pea on brain-derived neurotrophic factor (BDNF), a neuronal biomarker, in the prefrontal cortex and hippocampus. The results are shown in Figure 11B. The stress group exhibited a significant decrease in BDNF levels in both the prefrontal cortex and hippocampus. Treatment with the combined extract at doses of 250 and 500 mg/kg BW mitigated the decrease in BDNF levels in the prefrontal cortex. In the hippocampus, only the highest dose of the combined extract (500 mg/kg BW) significantly increased BDNF levels. The positive control drug groups—including diazepam, donepezil, and fluoxetine—significantly increased BDNF levels in both the prefrontal cortex and hippocampus.

### 3.6. Histological Changes

This study explored the positive effects of a combined extract from mulberry leaves and butterfly pea flowers on memory, focusing on changes in neuron density either through neurogenesis or reduced neurodegeneration. It investigated neuron density in the prefrontal cortex and various hippocampal regions (CA1, CA2, CA3, and the dentate gyrus) to better understand the underlying mechanism.

**Figure 12:**
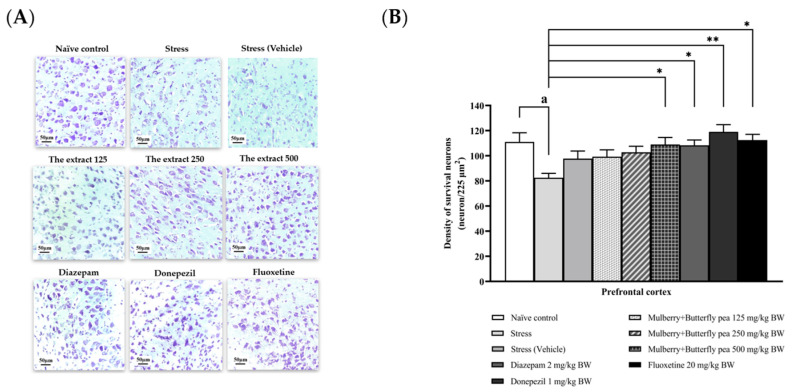
The effect of the extract of mulberry leaves and butterfly pea on the density of neurons in the frontal cortex. (**A**) Representative histological pictures showing neuron density in the prefrontal cortex. (**B**) Bar graph comparing neuron density in the prefrontal cortex. ^a^ *p* < 0.05 when compared with the naïve control group. *^,^ ** *p* < 0.05 and <0.01 when compared with stress group.

Figure 12A,B shows the effect of the combined extract of mulberry leaves and butterfly pea flowers on neuron density in the prefrontal cortex. The stress group exhibited a significant decrease in neuron density in this area, whereas the stress + vehicle group did not show a significant change in neuron density in the prefrontal cortex. However, this decrease was mitigated by donepezil and the highest dose (500 mg/kg BW) of the combined extract used in this study.

**Figure 13:**
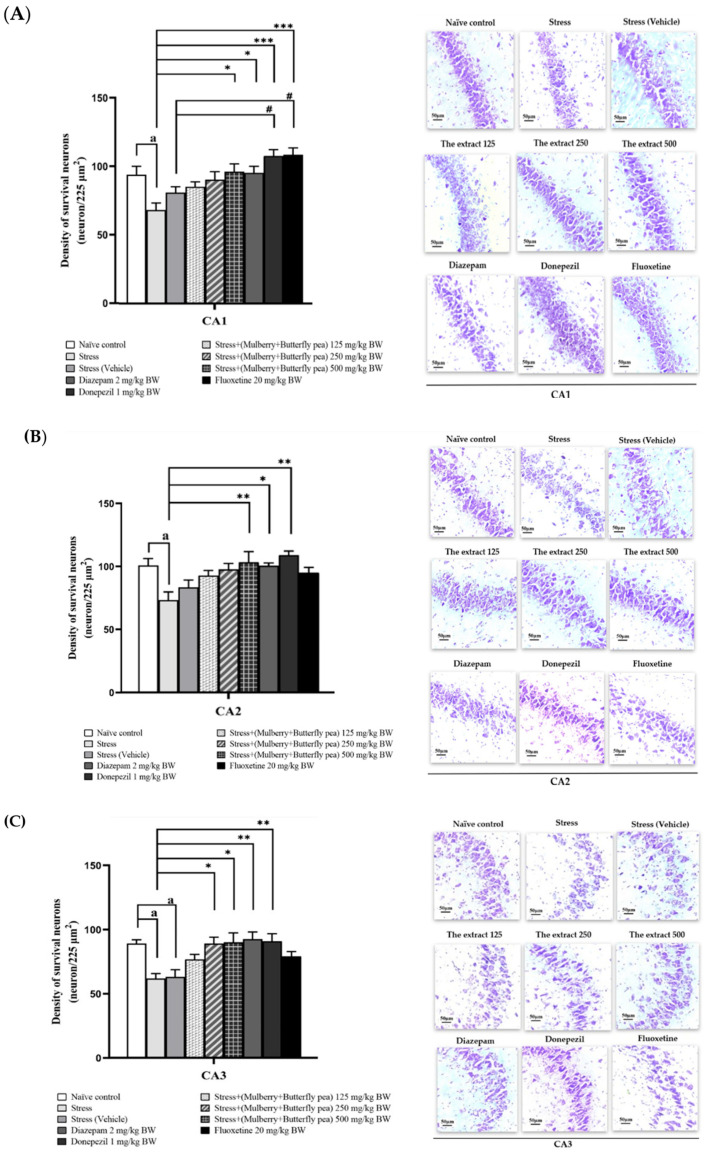

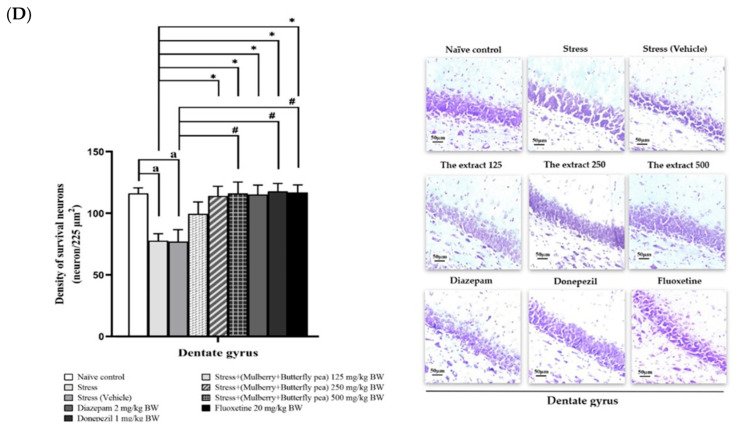
The effect of the extract of mulberry leaves and butterfly pea flowers on the density of neurons in the hippocampus. (**A**) Bar graph comparing and representative histological pictures showing neuron density in CA1, (**B**) CA2, (**C**) CA3, and (**D**) dentate gyrus of the hippocampus. Data are presented as mean ± SEM. ^a^ *p* < 0.05 when compared with naïve control group. *^,^ **^,^ *** *p* < 0.05, <0.01 and <0.001 when compared with stress group. ^#^ *p* < 0.05 when compared with stress + vehicle group.

Neuron density in the CA1, CA2, CA3, and dentate gyrus (DG) regions of the hippocampus is detailed in Figure 13A–D. Exposure to stress resulted in a significant decrease in neuron density across all hippocampal subregions. The stress + vehicle group did not show improvement in neuron density in CA1 and CA2, while a further reduction was observed in CA3. Administration of the combined extract at 125 mg/kg did not significantly affect neuron density in either the prefrontal cortex or hippocampus. In contrast, treatment with 250 mg/kg of the extract led to a significant increase in neuronal density in the CA3 and DG. Furthermore, both 500 mg/kg of the extract and donepezil effectively prevented neuron loss in CA1, CA3, and DG regions.

## 4. Discussion

The results obtained from this study show that the combined extract of mulberry leaves and butterfly pea flowers exhibits anti-insomnia, anxiolytic, antidepressant, and memory-enhancing effects in rats induced by stress. These functional ingredients also enhance brain-derived neurotrophic factor (BDNF) levels and neuron density in the prefrontal cortex and hippocampus. Additionally, they reduce oxidative stress, corticosterone, and interleukin-6 (IL-6) levels, as well as improve the functioning of the GABAergic, cholinergic, and monoaminergic systems in both brain regions.

The physiological stress response involves two main systems: the rapid sympatho-adreno-medullary (SAM) system and the slow response hypothalamic-pituitary-adrenal (HPA) axis. When stress occurs, the SAM system rapidly increases production and release of norepinephrine (NE) and epinephrine (E) [32]. Elevated NE concentrations in neural tissue generate oxidative stress via primary biochemical pathways. The enzymatic degradation of NE by monoamine oxidase (MAO) produces hydrogen peroxide as a metabolic byproduct [33]. Additionally, these processes increase mitochondrial oxygen consumption, thereby generating reactive oxygen species (ROS) via the respiratory chain. These oxidative processes manifest lipid peroxidation, evidenced by elevated malondialdehyde (MDA) concentrations in affected tissues. At the same time, ROS can involve the balancing of the antioxidant enzyme activity, including superoxide dismutase (SOD) and catalase (CAT) [34]. The resulting imbalance between ROS production and antioxidant enzyme capacity may contribute to the progression of neurodegenerative conditions [35]. This study demonstrated that the combined extract of mulberry leaves and butterfly pea flowers reduced MDA levels and increased SOD and CAT levels. Additionally, the extract decreased MAO activity, indicating a potential role in the regulation of catecholamine neurotransmitters and the mitigation of neurodegeneration. These experimental results are consistent with previous research showing that mulberry treatment enhanced memory function and reduced oxidative stress in the prefrontal cortex and hippocampus of rats with metabolic syndrome [36]. Moreover, a previous study has shown that the hydroalcoholic extract of butterfly pea flowers can prevent streptozotocin (STZ)-induced cognitive impairment in a dose-dependent manner by reducing oxidative stress and cholinesterase activity. This indicates its potential in protecting against neurodegenerative conditions where oxidative stress plays a key role [37].

Repetitive stress exposure leads to progressive dysfunction of the hypothalamic-pituitary-adrenal (HPA) axis, characterized by sustained elevation of glucocorticoid hormones such as cortisol in humans and corticosterone in rodents [38]. Excess cortisol, often resulting from repetitive stress, activates glucocorticoid receptors, which in turn boost cellular creation of ROS such as superoxide and hydrogen peroxide. These ROS can damage lipids, proteins, and DNA [39,40]. Additionally, a number of studies have indeed shown that flavonoids and anthocyanins can help reduce glucocorticoid levels. These compounds are potent antioxidants that can mitigate the effects of oxidative stress, which is often associated with elevated cortisol levels [41,42]. Interestingly, our results revealed that all doses of the combined extract alleviated the corticosterone level in both the prefrontal cortex and hippocampus. Therefore, in this study, the primary mechanism of the combined extract may involve reducing corticosterone levels.

Moreover, excess corticosterone has significant immunological consequences, including altered lymphocyte populations and functions, which result in lymphopenia and a reduced capacity for specific immune responses and the formation of immunological memory [43]. Additionally, activation of inflammatory pathways is frequently observed in stress-related disorders, as indicated by elevated levels of pro-inflammatory cytokines such as interleukin-1β (IL-1β), interleukin-6 (IL-6), and tumor necrosis factor α (TNF-α) [44]. This study found that the combined extract significantly reduced IL-6 levels in the stress-exposed rats. These findings are consistent with previous research, which demonstrated a decrease in IL-6 levels in stressed mice following the administration of mulberry extract [45].

According to the brain-derived neurotrophic factor (BDNF), a neurotrophin, it is crucial for the survival and differentiation of neurons during development [46]. Numerous studies have directly associated BDNF with learning and memory. For instance, BDNF may act as a mediator for the plastic changes that support both spatial and recognition memory processes [47,48]. Therefore, our study suggests that the memory-enhancing effects of the combined extract of mulberry leaves and butterfly pea flowers may be partly attributed to increased BDNF levels, which promote neuron survival and improve neurotransmitter function. These effects enhance monoamine and acetylcholine activity, contributing to improved mood and memory. Moreover, the combined extracts improved escape latency in the Morris water maze test. The reduced escape latency involved the enhanced spatial learning and memory encoding [49]. Memory formation consists of three stages: encoding, storage, and retrieval. Faster escape latency indicates enhanced encoding, the initial phase of memory. In contrast, the probe task in the Morris water maze assesses retention, which happens after memory consolidation and during the retrieval stage [50]. Different brain regions are involved in encoding and retrieving memories. For instance, the hippocampus encodes items, while the parietal and fusiform regions handle location and time information. Retrieval also involves specific areas, with the inferior frontal and parietal regions for items and the frontal and anterior cingulate cortices for location [51]. Therefore, the combined extract may influence the encoding stage of memory more than the retrieval stage in the first week. In addition, acetylcholine (ACh) also plays a critical role in learning and memory. An elevation in central ACh levels activated by the suppression of acetylcholinesterase (AChE) can enhance memory ability and comprehensively improve brain function [52]. Our results are also consistent with the above-mentioned findings. The combined extract of mulberry leaves and butterfly pea flowers can mitigate the level of AChE. Additionally, this study found that stressed rats experienced a loss of pyramidal neurons in the prefrontal cortex and hippocampus, which is associated with previous studies showing that the neuron density loss in the stressed rats [53,54].

The GABAergic system is involved in sleep regulation. By modulating GABA levels, GABA-T indirectly influences the sedative effects associated with GABA [55]. When GABA levels are high, it enhances inhibitory signals in the brain, leading to sedation and relaxation [56]. In this study, GABA-T levels in serum did not significantly decrease at any dose of the combined extract when compared to the stress group. Based on our experimental results, serum GABA-T measurements may be compromised by the rapid metabolism of GABA-T in blood circulation, potentially leading to inaccurate readings. Therefore, future studies should investigate alternative biomarkers related to sleep regulation, such as melatonin levels. Melatonin is a hormone produced by the pineal gland in the brain that plays a crucial role in regulating the body’s sleep-wake cycle [57]. Measuring the melatonin levels can help understand sleep problems and ways to improve sleep quality. Additionally, this study recommends that analyzing electroencephalographic (EEG) activity during sleep could provide a more precise explanation of the experimental results. This is particularly relevant, as slow-wave sleep (SWS) has been closely associated with mental health issues, notably depression and cognitive dysfunction [58]. Individuals with depression frequently exhibit reduced SWS, and such a deficiency may contribute to both the development and persistence of depressive symptoms [59]. According to stress-related disorders, our data also improved anxiety and depression in the elevated plus maze and forced swimming test, respectively. The results reveal that the combined extract exhibits anxiolytic and antidepressant-like behavior in the second week.

Given the anti-insomnia, anxiolytic, memory-enhancing, and antidepressant properties—along with the anti-inflammatory and antioxidant capacities—of polyphenols, particularly anthocyanins and rutin found in mulberry leaves and butterfly pea flowers, it is proposed that their cognitive benefits may be partly attributable to the actions of these absorbed polyphenolic compounds [18,21]. Furthermore, it has been suggested that the cognitive-enhancing effects of mulberry leaves and butterfly pea flowers may occur partly due to the influence of these absorbed polyphenols. The combined extract used in this study, prepared from mulberry leaves and butterfly pea flowers, contains high levels of anthocyanins, particularly peonidin-3-O-glucoside and cyanidin-3-rutinoside, which are recognized for their potent antioxidant and anti-inflammatory activities [60]. Cyanidin derivatives are also reported to possess antidepressant and anxiolytic effects [61,62]. Furthermore, the extract contains significant levels of gallic acid and rutin, two well-characterized polyphenols known for their free radical scavenging capabilities and their potential to combat oxidative stress and inflammation [63]. Based on these findings, it is proposed that the observed mental health benefits in this study may be attributed, at least in part, to the synergistic action of these polyphenolic compounds. However, the gut-brain axis may play a significant role in the underlying mechanisms of the combined extract effect. Since the combined extract of mulberry leaves and butterfly pea flowers predominantly reaches the large intestine, where the gut microbiota resides, polyphenols in this extract may modulate the gut-brain axis, contributing to enhanced brain protection and function.

## 5. Conclusions

The results indicated that the functional ingredients from mulberry leaves and butterfly pea flowers exhibited sedative, anxiolytic, cognitive-enhancing, and antidepressant effects. The potential mechanisms likely operate through reductions in corticosterone levels and decreased neuronal death in the prefrontal cortex and hippocampus. Additionally, the functional ingredients reduced the activity of AChE, MAO, and GABA-T enzymes, while decreasing MDA levels and increasing scavenging enzyme and BDNF levels. Therefore, the combined extract demonstrates promising potential for neuroprotective applications. However, the use of butterfly pea flowers should take into consideration their safety and non-toxicity to the human body. Although they are generally permitted for consumption in many regions, children and pregnant women should take precautions and consume them in moderation until more comprehensive safety information becomes available. In addition, further research investigating specific mechanisms and clinical trials remains necessary in order to validate these findings.

## Figures and Tables

**Table 1:** Groups of rats receive experimental treatments.

Treatment Group	Experimental Details
Group I Naïve control	Rats not receiving any treatment
Group II Stress	Rats were immobilized for 6 h to stress, and they were not given any treatment.
Group III Stress + Vehicle	Rats were immobilized for 6 h to stress after they were given distilled water.
Group IV Stress + the combined extract of mulberry leaves and butterfly pea flowers 125 mg/kg BW	Rats were immobilized for 6 h to stress after they were given the combined extract at a dose of 125 mg/kg BW.
Group V Stress + the combined extract of mulberry leaves and butterfly pea flowers 250 mg/kg BW	Rats were immobilized for 6 h to stress after they were given the combined extract at a dose of 250 mg/kg BW.
Group VI Stress + combined extract of mulberry leaves and butterfly pea flowers 500 mg/kg BW	Rats were immobilized for 6 h to stress after they were given the combined extract at a dose of 500 mg/kg BW.
Group VII-IX Stress+ Positive control	Rats were immobilized for 6 h to stress after they were given diazepam (2 mg/kg BW) (GPO^®^, Bangkok, Thailand) to assess its ability to reduce sedative effect and anxiety, while donepezil (1 kg/kg BW) (Tonizep^®^, Bangkok, Thailand) and fluoxetine (20 mg/kg BW) (FULOX^®^, Bangkok, Thailand) were also tested for their effects.

**Table 2:** Oxidative stress markers, including malondialdehyde (MDA) level and the activities of superoxide dismutase (SOD) and catalase (CAT) enzymes in the prefrontal cortex of various treatment groups. Data are presented as mean ± SEM (*n* = 6/group). ^a, aa, aaa^ *p* < 0.05 when compared with naïve control group. *^,^ ** *p* < 0.05, < 0.01 when compared with stress group. ^#^ *p* < 0.05 when compared with stress + vehicle group.

Groups		Prefrontal Cortex	
	Malondialdehyde Level (nmol/mg Protein)	Superoxide Dismutase Activity (U/mg Protein)	Catalase Activity (U/mg Protein)
Naïve control	0.072 ± 0.02	36.07 ± 1.66	9.22 ± 0.72
Stress	0.158 ± 0.01 ^aaa^	25.56 ± 1.18 ^aa^	3.61 ± 0.93 ^a^
Stress + Vehicle	0.149 ± 0.01 ^a^	28.59 ± 1.84	4.07 ± 0.41
Stress + The combined extract of mulberry leaves and butterfly pea flowers 125 mg/kg BW	0.010 ± 0.01	31.73 ± 1.87	7.25 ± 1.83
Stress + The combined extract of mulberry leaves and butterfly pea flowers 250 mg/kg BW	0.079 ± 0.02 **	34.19 ± 0.97 *	9.02 ± 0.79 *
Stress + The combined extract of mulberry leaves and butterfly pea flowers 500 mg/kg BW	0.077 ± 0.01 **^, #^	36.08 ± 1.96 **	10.39 ± 1.48 **
Stress + Diazepam	0.095 ± 0.01 *	32.89 ± 1.17 *	8.93 ± 1.06
Stress + Donepezil	0.090 ± 0.01 *	34.00 ± 1.89 *	10.53 ± 1.49 **
Stress + Fluoxetine	0.083 ± 0.01 **	34.37 ± 2.27	9.74 ± 1.09 *

**Table 3:** Oxidative stress markers, including malondialdehyde (MDA) level and the activities of superoxide dismutase (SOD) and catalase (CAT) enzymes in the hippocampus of various treatment groups. Data are presented as mean ± SEM (*n* = 6/group). ^a, aa^ *p* < 0.05 when compared with naïve control group. *^,^ **^,^ *** *p* < 0.05, <0.01 and <0.001 when compared with stress group. ^#^ *p* < 0.05 compared with stress + vehicle group.

Groups		Hippocampus	
	Malondialdehyde Level (nmol/mg Protein)	Superoxide Dismutase Activity (U/mg Protein)	Catalase Activity (U/mg Protein)
Naïve control	0.081 ± 0.01	36.53 ± 0.85	11.54 ± 1.48
Stress	0.154 ± 0.01 ^aa^	25.45 ± 3.73 ^aa^	4.95 ± 0.56 ^a^
Stress + Vehicle	0.146 ± 0.03 ^a^	26.30 ± 1.21^a^	6.16 ± 1.45
Stress + The combined extract of mulberry leaves and butterfly pea flowers 125 mg/kg BW	0.091 ± 0.02 **	36.00 ± 1.15 **	9.77 ± 1.19
Stress + The combined extract of mulberry leaves and butterfly pea flowers 250 mg/kg BW	0.083 ± 0.01 **	37.08 ± 2.13 ***^, #^	10.2 ± 1.97
Stress + The combined extract of mulberry leaves and butterfly pea flowers 500 mg/kg BW	0.083 ± 0.01 **^, #^	38.00 ± 0.90 ***^, #^	11.30 ± 1.57 *
Stress + Diazepam	0.088 ± 0.01 **	34.24 ± 1.08 *	8.23 ± 0.90
Stress + Donepezil	0.086 ± 0.01 **	35.57 ± 1.28 **	11.42 ± 1.08 *
Stress + Fluoxetine	0.089 ± 0.01 **	35.64 ± 1.15 **	8.63 ± 1.26

## Data Availability

The data used in this study can be obtained upon request from the corresponding author. However, the data are not publicly accessible due to trade secrecy and the ongoing patent application process.

## Supplementary Materials

The following supporting information can be downloaded at: https://www.mdpi.com/article/10.3390/life15081308/s1. Title: The fingerprint of the combine extract of mulberry leaves and butterfly pea flowers.
