# Supplementary material for: The Functional Ingredients of the Combined Extract of Mulberry Leaves and Butterfly Pea Flowers Improve Insomnia, Anxiolytic, Memory-Enhancing, and Antidepressant-like Activities in Stress-Exposed Rats
Source: Life (Basel). 2025 Aug 18;15(8):1308. doi: 10.3390/life15081308 (PMC12387157; doi:10.3390/life15081308)
Supplement: Supplementary file 1 [file life-15-01308-s001.zip › life-3771429-supplementary.pdf]

The report of the analysis of mulberry leaves and butterfly pea flowers extract using High Performance Liquid Chromatography (HPLC).

**Sample**	**The combined extract of mulberry leaves and butterfly pea flowers**
**Date**	29 February 2024

1. **Chemicals**

**Name of compound**	**Condensed Formular**	**Structural Formular**
**Reference standard:**		
Gallic acid	$C_7H_6O_5$	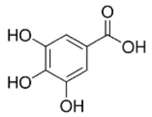
Quercetin	$C_{15}H_{10}O_7$	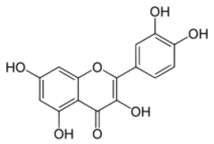
Kaempferol	$C_{15}H_{10}O_6$	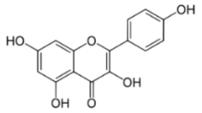
Chlorogenic acid	$C_{16}H_{18}O_9$	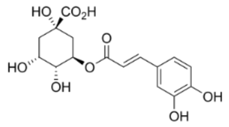
Caffeic acid	$C_9H_8O_4$	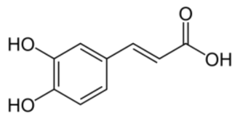
Ferulic acid	$C_{10}H_{10}O_4$	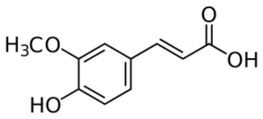
p-Coumaric acid	$C_9H_8O_3$	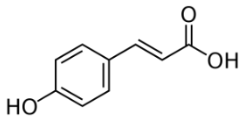

Catechin	$C_{15}H_{14}O_6$	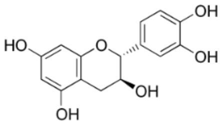
Ellagic acid	$C_{14}H_6O_8$	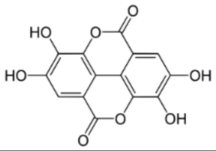
**Mobile phase:**		
Formic acid	CH_2O_2	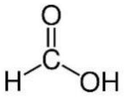
Acetonitrile	CH_3CN	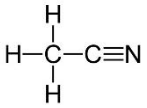
Deionized water (DI)	H_2O	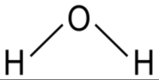

2. Analytical instrument

- The HPLC system, Waters (USA) consisted of an In-line degasser AF, a Waters 515 HPLC pump (pump control module II), a Rheodyne injector with a sample loop of 20 μL, a Waters 2998 photodiode array detector.
- Vortex mixer (50 Hz), Scientific Industries, INC (USA)
- Micropipette 1000 μL, Gilson (UAS)
- Precision balance (Sartorius, Germany)
- HPLC columns, Poroshell 120 EC-C_{18} (250×4.6 mm id, 4.0 μm), Agilent Technologies (USA)
- UHPLC Guard Column Poroshell 120 EC-C18 column (5×4.6 mm id, 4.0 μm), Agilent Technologies (USA)
- 13 mm nylon membrane filters, 0.22 micrometer pore size, CNW (China)

3. The procedure

3.1 Optimal conditions for HPLC system for the quantitative analysis of the combined extract.

Mobile phase:	Acetonitrile (A), 0.1% Formic acid (B)
Flow rate measurement:	Gradient
Flow rate:	1 ml/min
The injected volume:	20 microliters
Wavelength of detection:	254, 275, 280, 310, 320 and 370 nanometers

Time (minute)	%B
0	90
10	75
20	40
30	30
35	20

3.2 Preparation of the sample

A 0.050 g sample was weighed into a 1.5 mL microcentrifuge tube. Subsequently, 1 mL of slightly acidic methanol (0.5% HCl in methanol) was added to the microcentrifuge tube. The tube was wrapped in aluminum foil, vortexed for 1 minute, and then sonicated for 20 minutes. The vortexed sample was centrifuged at 10,000 rpm at 4°C for 20 minutes. The supernatant was filtered through a 0.22 µm nylon membrane filter prior to injection into the HPLC system for further analysis.

4. Results

4.1 The overlay of standard and sample chromatograms

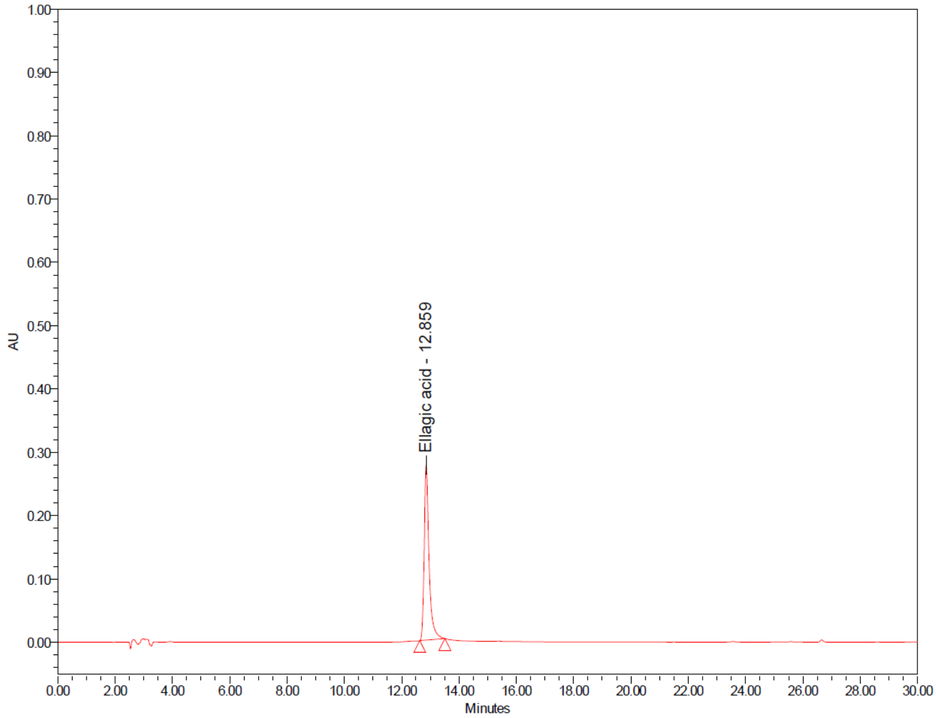

Figure 1 Chromatogram of a standard solution of ellagic acid at a concentration of 40 micrograms per milliliter at a wavelength of 254 nanometers.

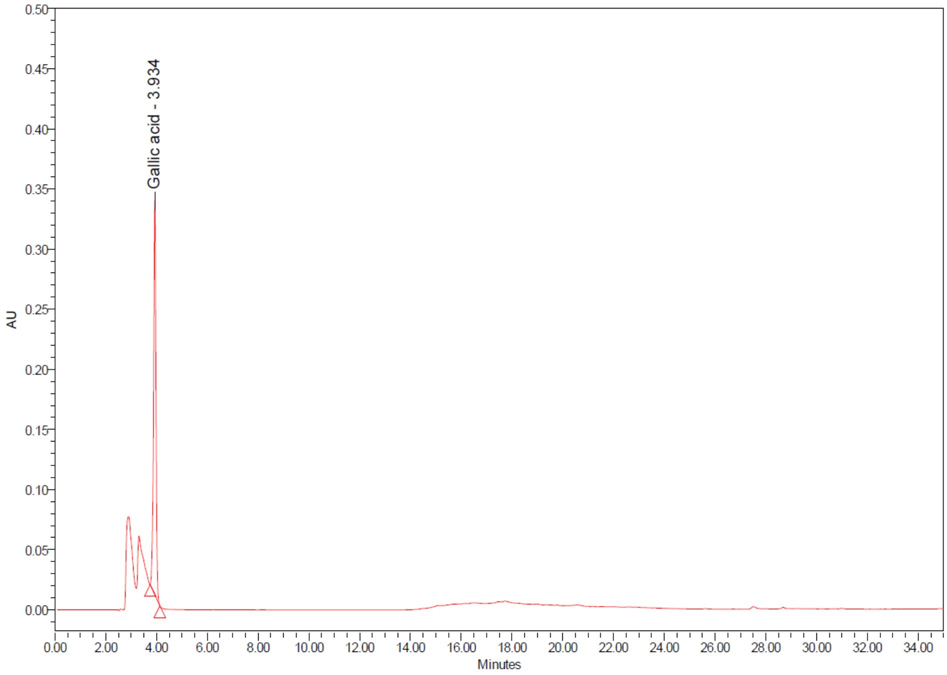

Figure 2 Chromatogram of a standard solution of Gallic acid at a concentration of 80 micrograms per milliliter at a wavelength of 275 nanometers.

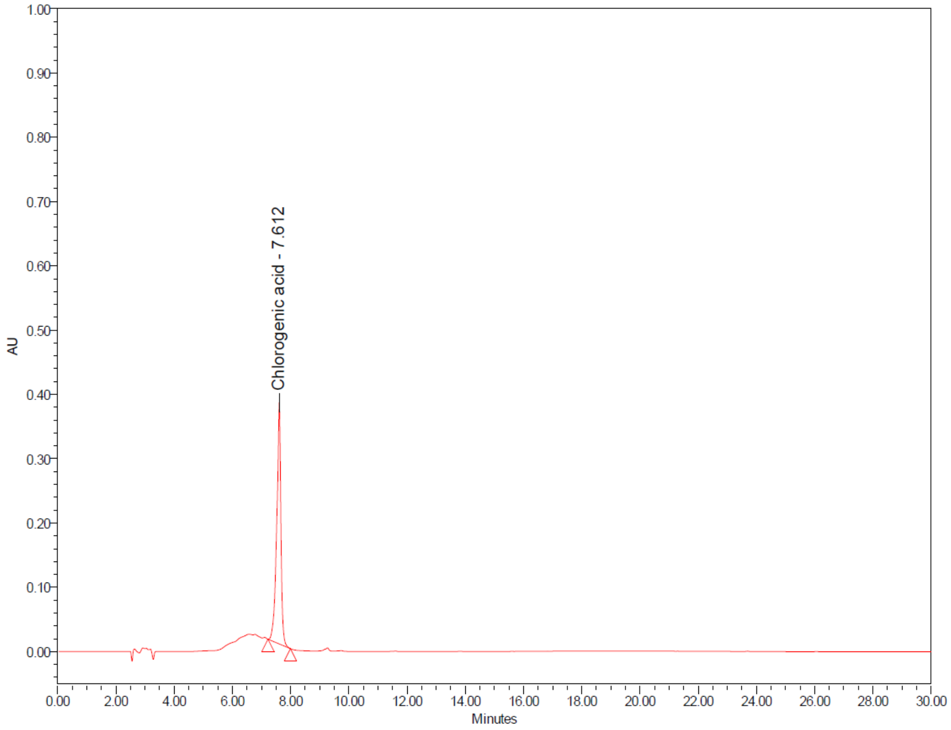

Figure 3 Chromatogram of a standard solution of Chlorogenic acid at a concentration of 100 micrograms per milliliter at a wavelength of 320 nanometers.

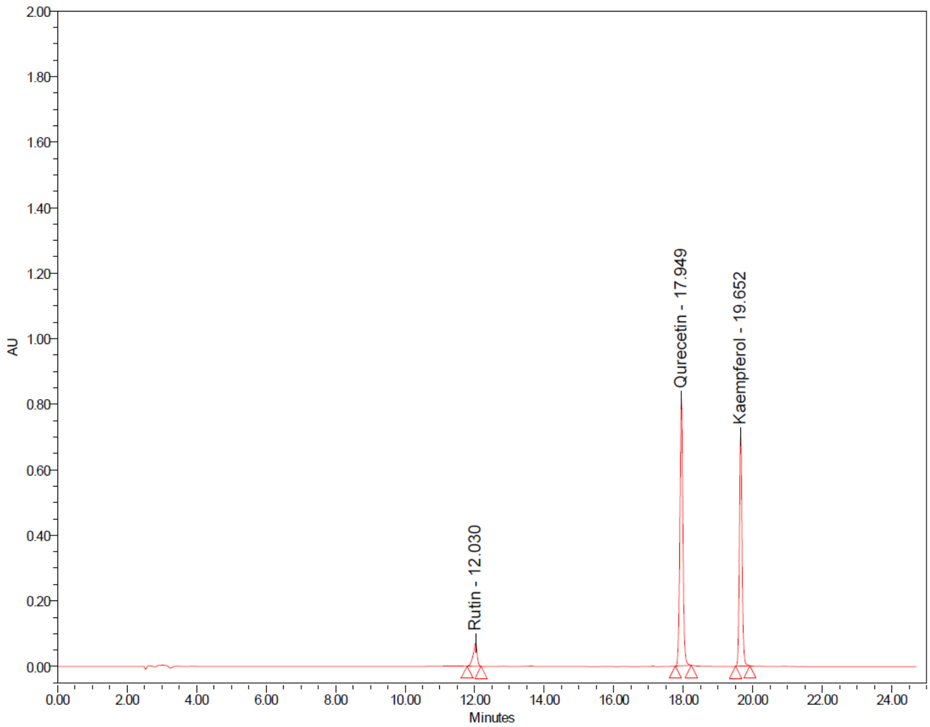

Figure 4 Chromatogram of a standard solution of Rutin, Quercetin and Kaempferol at a concentration of 40 micrograms per milliliter at a wavelength of 370 nanometers.

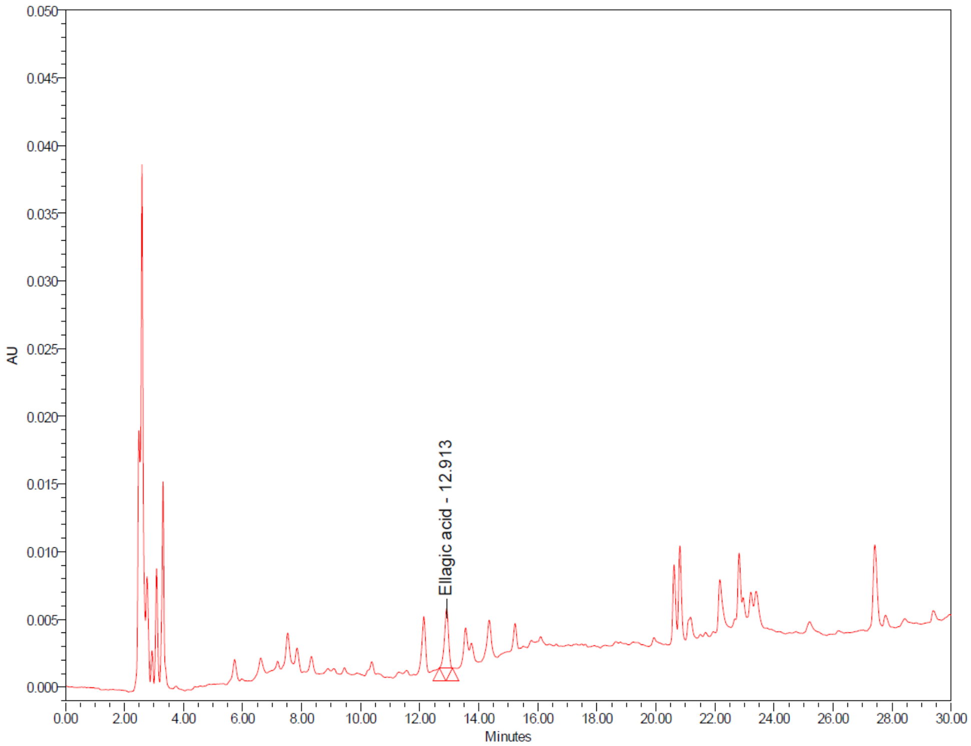

Figure 5 Chromatogram of the mulberry leaves extract at a concentration of 50 micrograms per milliliter at a wavelength of 254 nanometers.

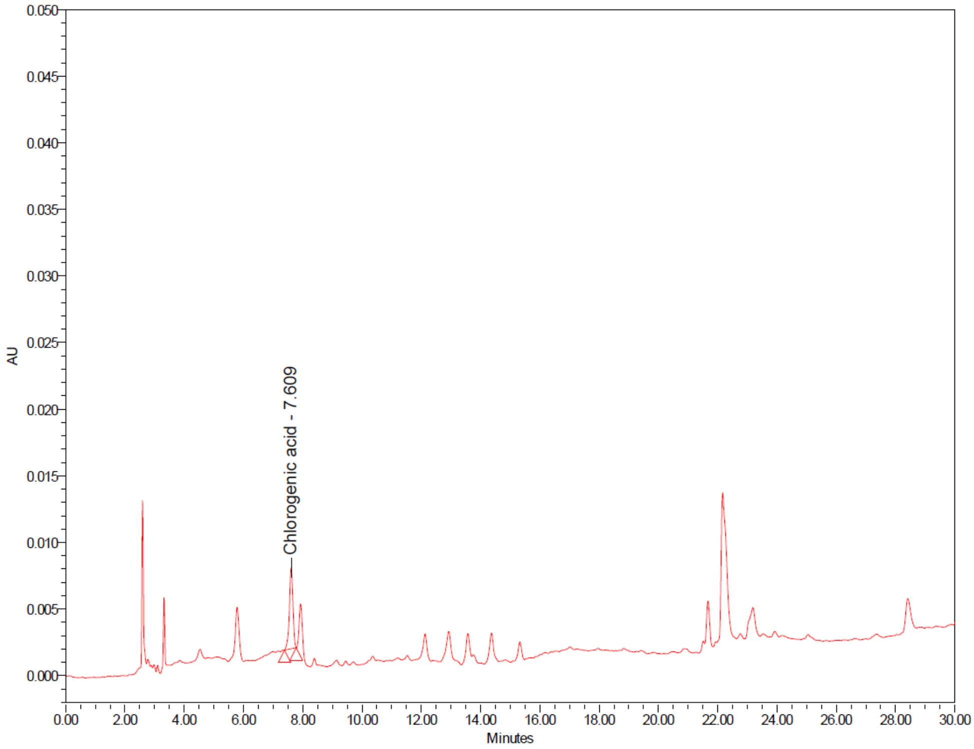

Figure 6 Chromatogram of the mulberry leaves extract at a concentration of 50 micrograms per milliliter at a wavelength of 320 nanometers.

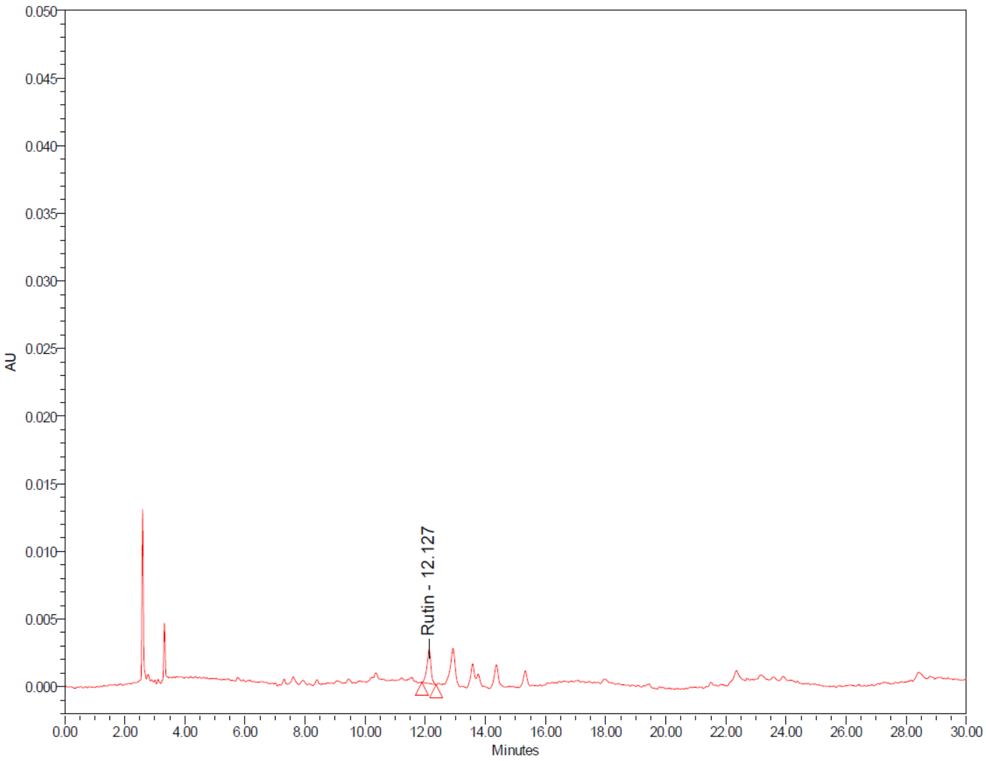

Figure 7 Chromatogram of the mulberry leaves extract at a concentration of 50 micrograms per milliliter at a wavelength of 370 nanometers.

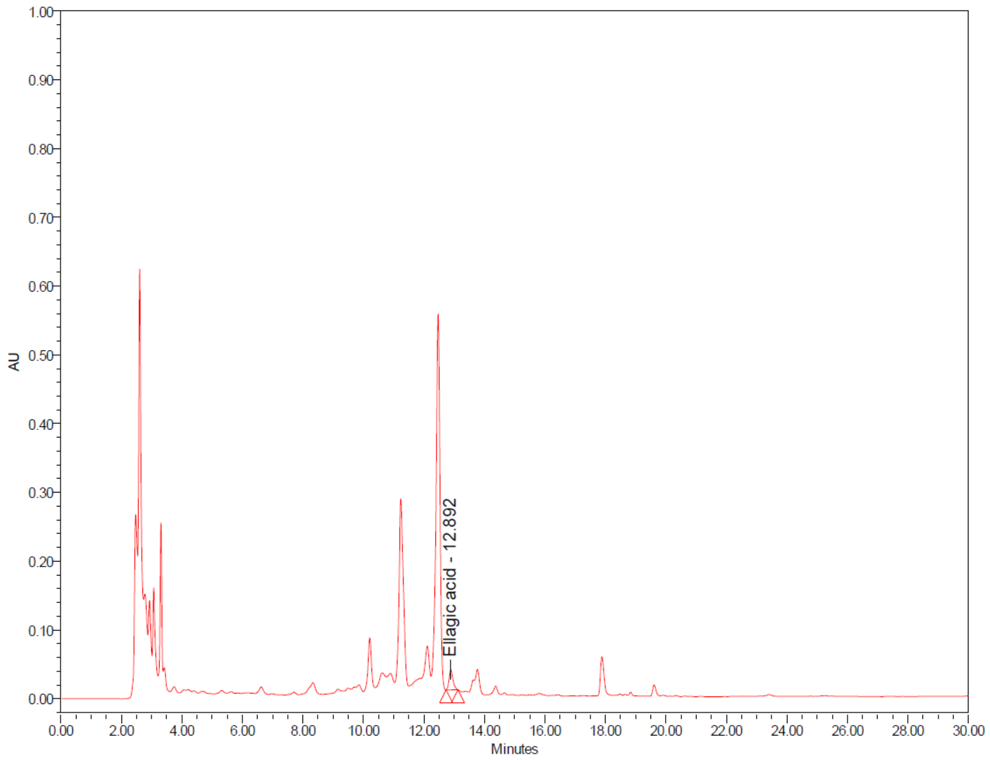

Figure 8 Chromatogram of the butterfly pea flowers extract at a concentration of 50 micrograms per milliliter at a wavelength of 254 nanometers.

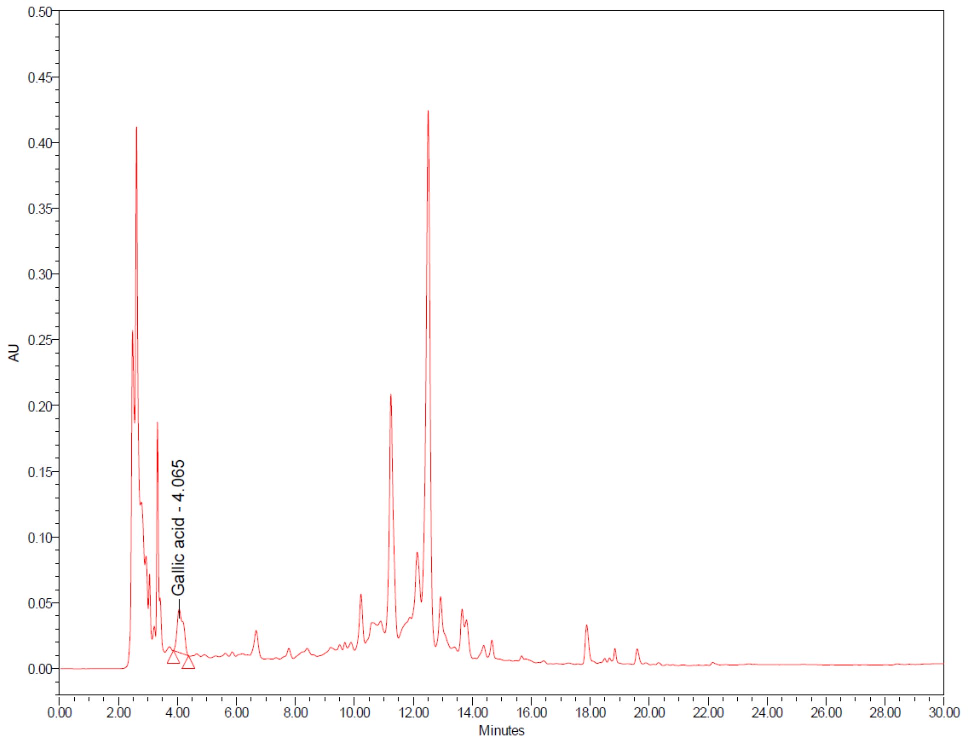

Figure 9 Chromatogram of the butterfly pea flowers extract at a concentration of 50 micrograms per milliliter at a wavelength of 275 nanometers.

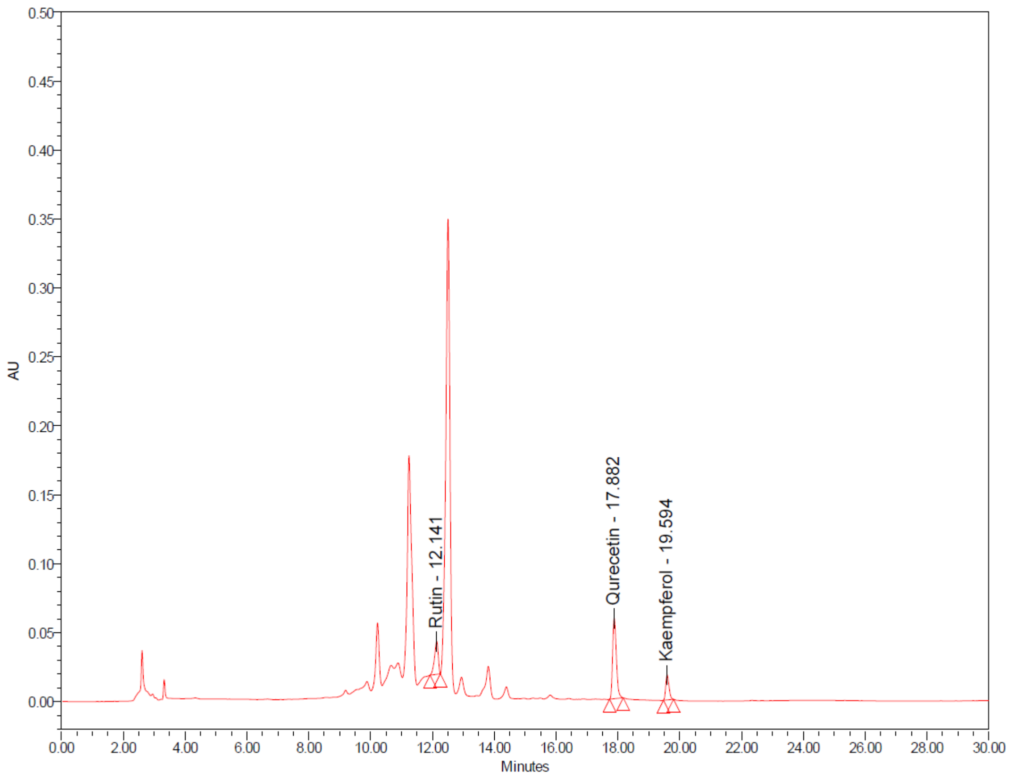

Figure 10 Chromatogram of the butterfly pea flowers extract at a concentration of 50 micrograms per milliliter at a wavelength of 370 nanometers.

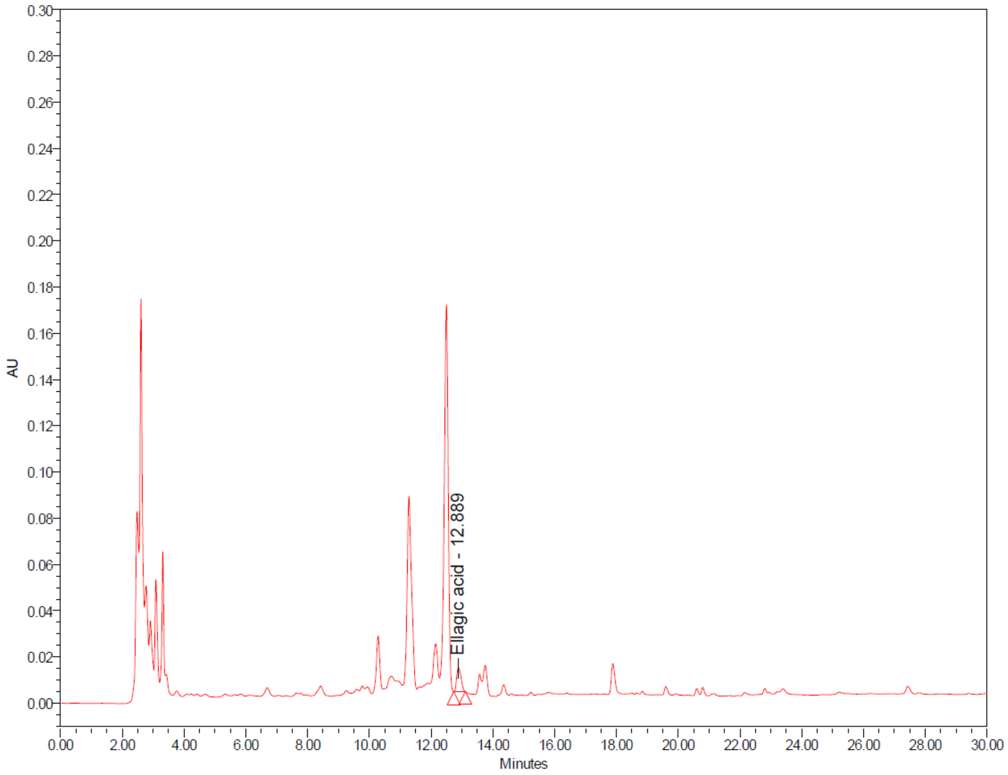

Figure 11 Chromatogram of the combined extract of mulberry leaves and butterfly pea flowers (3:1) at a concentration of 50 micrograms per milliliter at a wavelength of 254 nanometers.

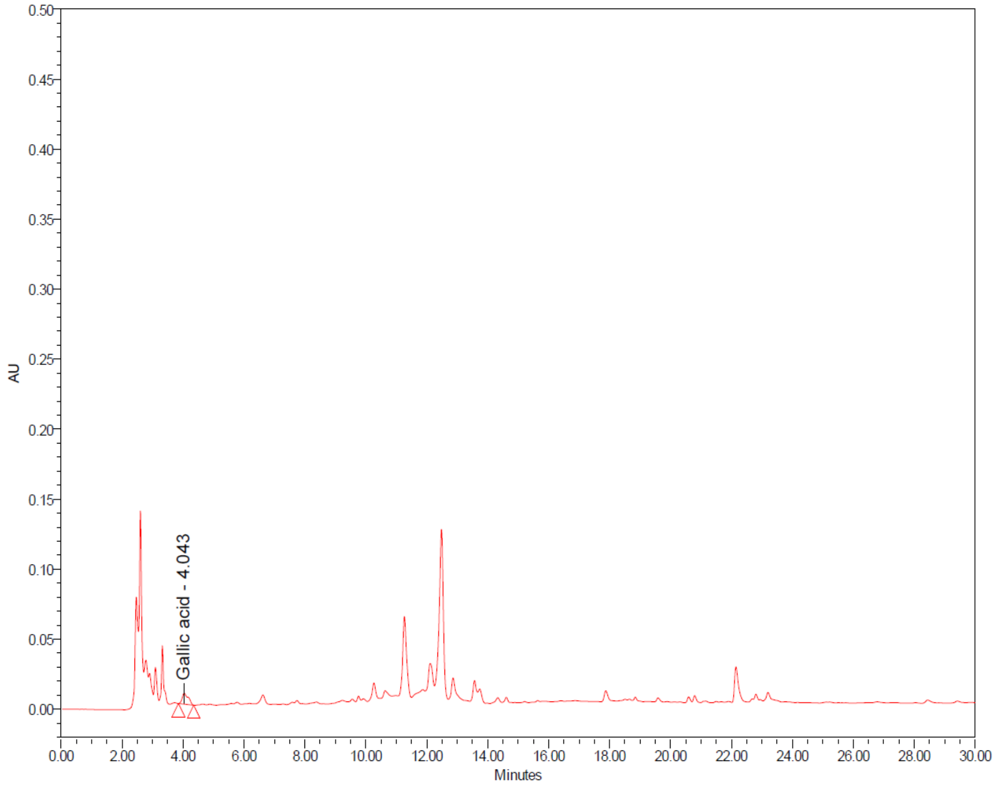

Figure 12 Chromatogram of the combined extract of mulberry leaves and butterfly pea flowers (3:1) at a concentration of 50 micrograms per milliliter at a wavelength of 275 nanometers.

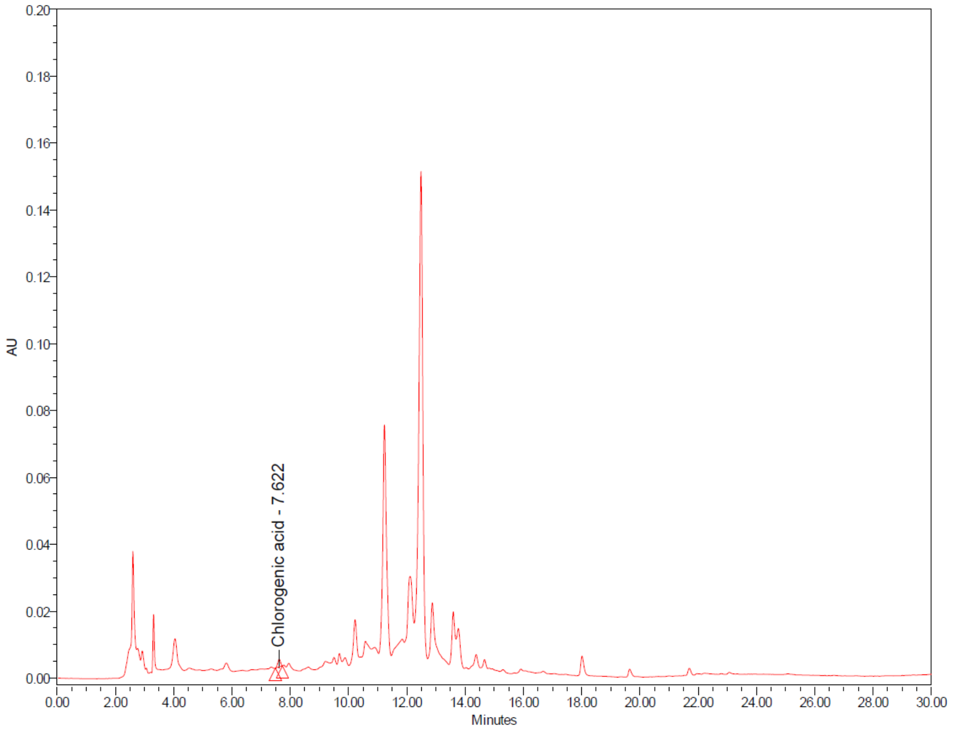

Figure 13 Chromatogram of the combined extract of mulberry leaves and butterfly pea flowers (3:1) at a concentration of 50 micrograms per milliliter at a wavelength of 320 nanometers.

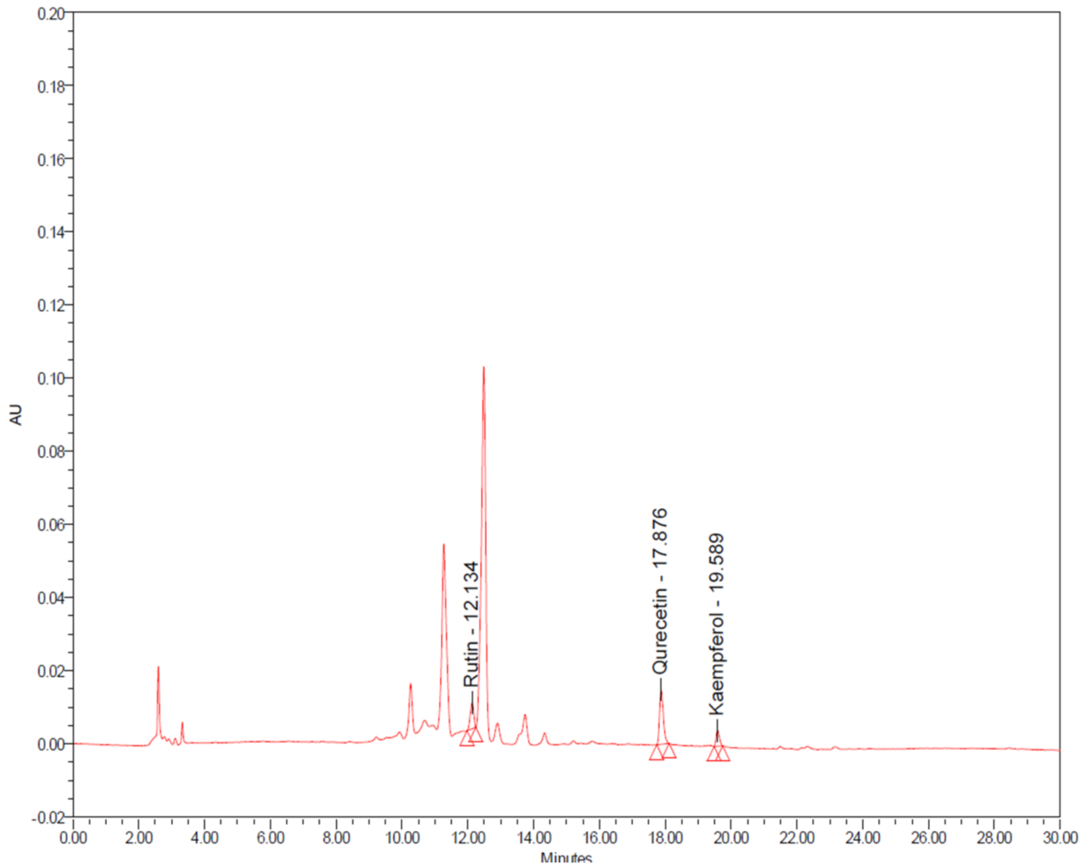

Figure 14 Chromatogram of the combined extract of mulberry leaves and butterfly pea flowers (3:1) at a concentration of 50 micrograms per milliliter at a wavelength of 370 nanometers.

4.2 linear equation

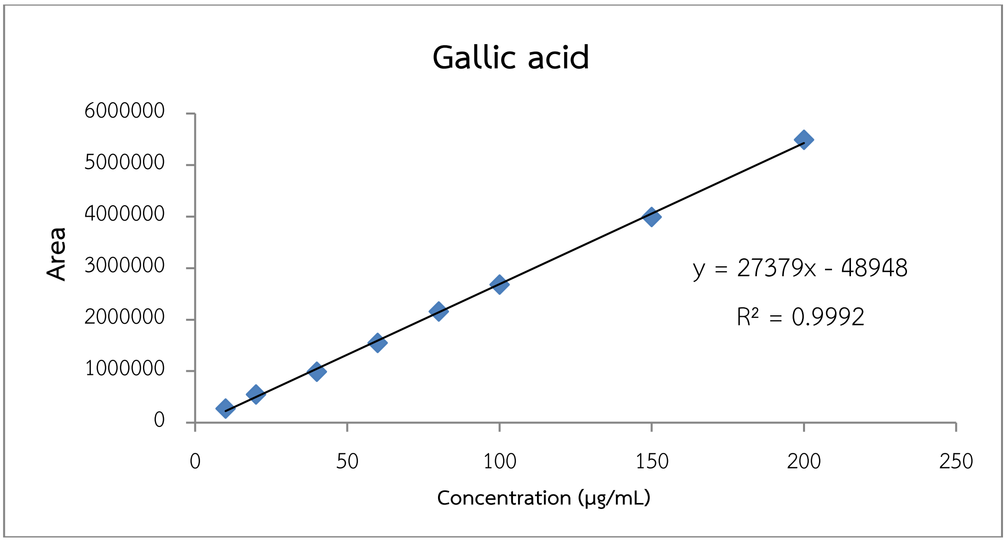

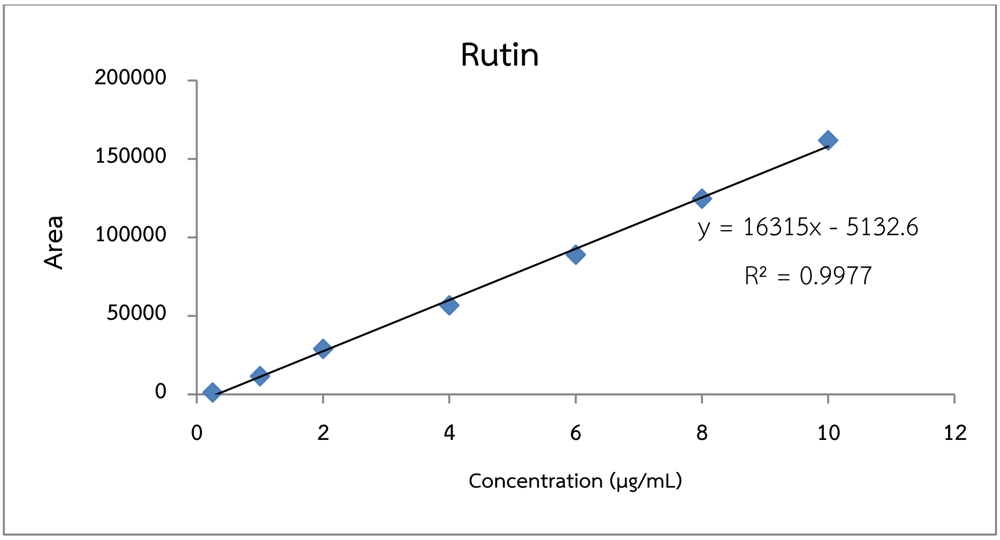

Rutin
Area
200000
150000
100000
50000
0
0
2
4
6
8
10
12
Concentration (µg/mL)
y = 16315x - 5132.6
R² = 0.9977

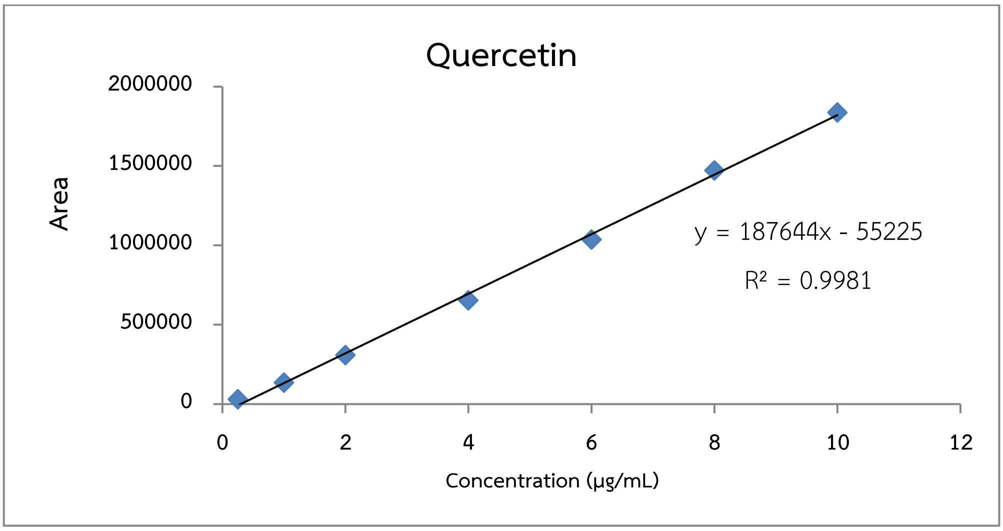

Quercetin
Area
2000000
1500000
1000000
500000
0
0
2
4
6
8
10
12
Concentration (µg/mL)
y = 187644x - 55225
R² = 0.9981

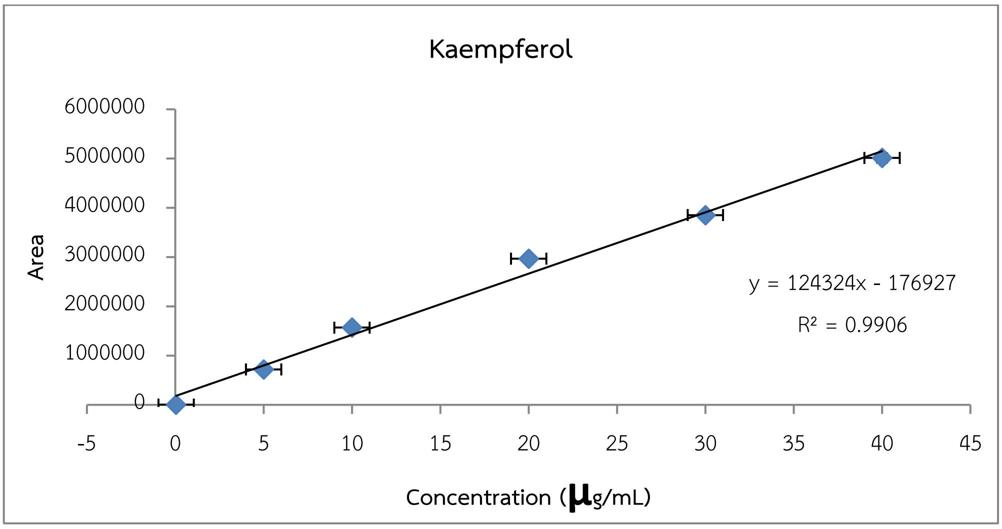

Kaempferol
Area
6000000
5000000
4000000
3000000
2000000
1000000
0
-5
0
5
10
15
20
25
30
35
40
45
Concentration (µg/mL)
y = 124324x - 176927
R² = 0.9906

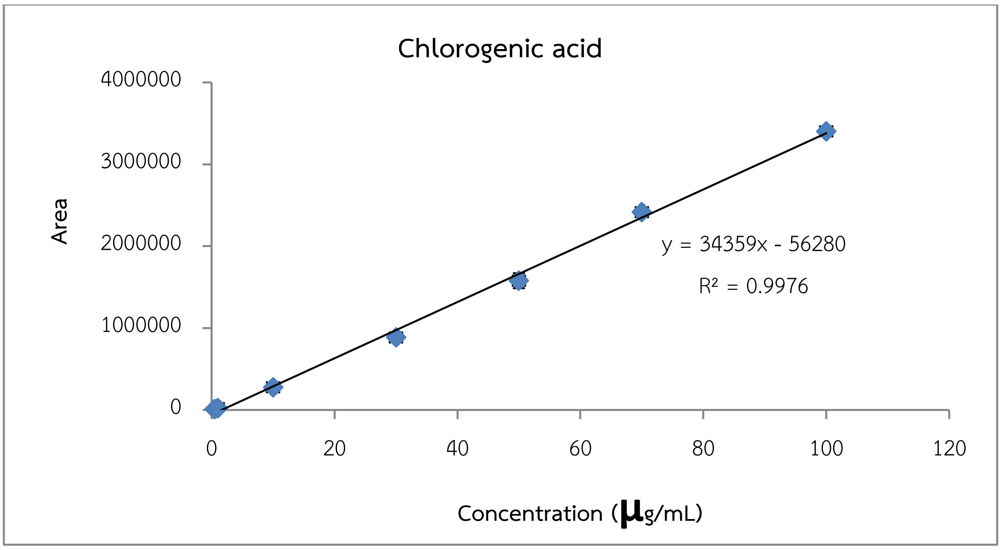
Chlorogenic acid
4000000
3000000
2000000
1000000
0
Area
0
20
40
60
80
100
120
y = 34359x - 56280
R² = 0.9976
Concentration (µg/mL)

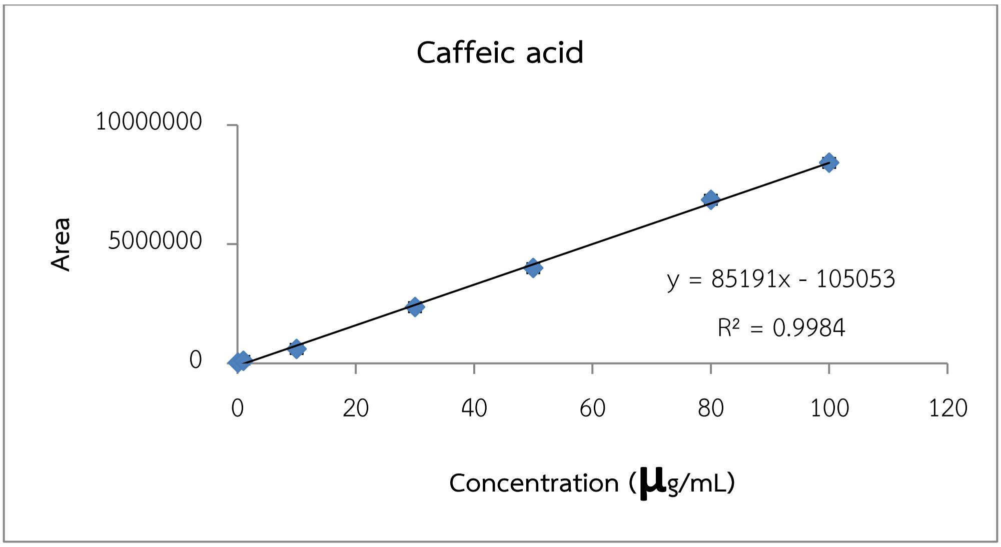
Caffeic acid
10000000
5000000
0
Area
0
20
40
60
80
100
120
y = 85191x - 105053
R² = 0.9984
Concentration (µg/mL)

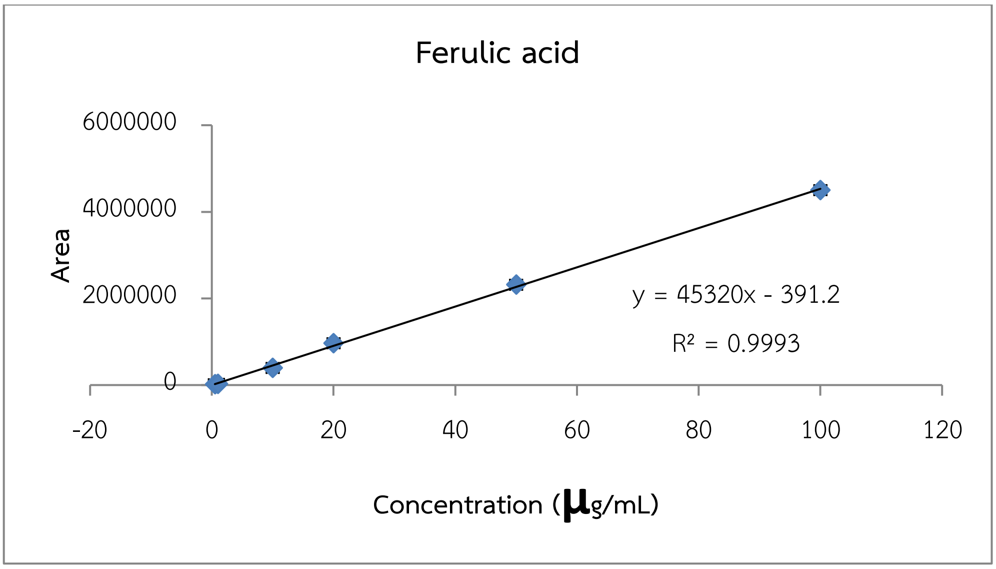
Ferulic acid
6000000
4000000
2000000
0
Area
-20
0
20
40
60
80
100
120
y = 45320x - 391.2
R² = 0.9993
Concentration (µg/mL)

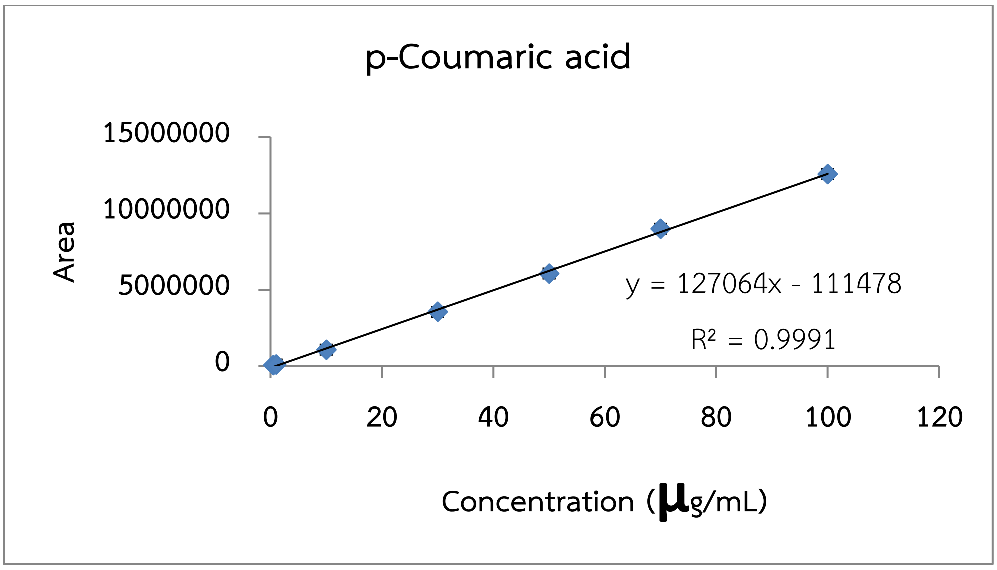
p-Coumaric acid
15000000
10000000
5000000
0
Area
0
20
40
60
80
100
120
y = 127064x - 111478
R² = 0.9991
Concentration (μg/mL)

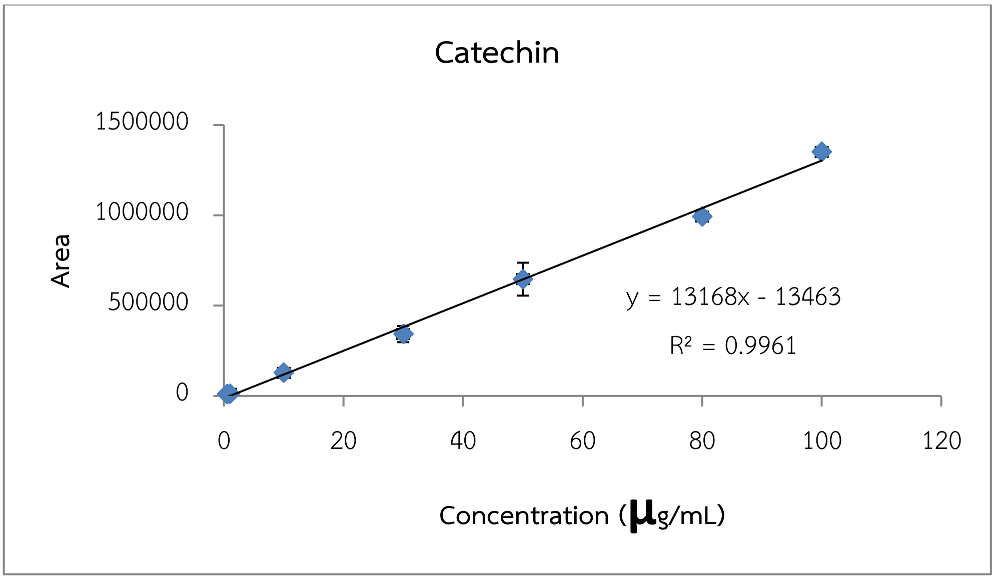
Catechin
1500000
1000000
500000
0
Area
0
20
40
60
80
100
120
y = 13168x - 13463
R² = 0.9961
Concentration (μg/mL)

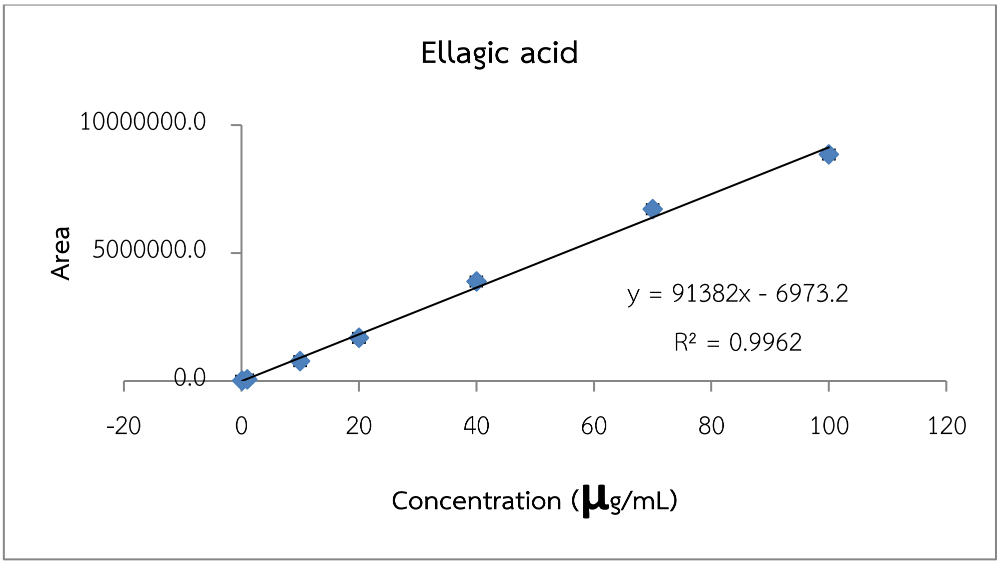
Ellagic acid
10000000.0
5000000.0
0.0
Area
-20
0
20
40
60
80
100
120
y = 91382x - 6973.2
R² = 0.9962
Concentration (μg/mL)

Table 1 Linear regression equations and correlation coefficients

Compound	Linear regression equations	Range of linearity (μg/mL)	correlation coefficients (r^2)
Gallic acid	y = 27379x – 48948	10–1000	0.9992
Rutin	y = 16315x – 5132.6	0.25–10	0.9977
Quercetin	y = 187644x – 55225	0.25–10	0.9981
Kaempferol	y = 124324x – 176927	0.05–40	0.9906
Chlorogenic acid	y = 34359x – 56280	0.5–100	0.9976
Caffeic acid	y = 85191x – 10553	0.1–100	0.9984
Ferulic acid	y = 45320x – 391.2	0.5–100	0.9993
p-Coumaric acid	y = 127064x – 111478	0.5–100	0.9991
Catechin	y = 13168x – 13463	0.5–100	0.9961
Ellagic acid	y = 91382x – 6973.2	0.1–100	0.9962

4.3 Analytical sensitivity

The lowest amount of analyte in a sample that can be quantitatively determined with acceptable precision and accuracy, with a signal-to-noise ratio of at least 10, is referred to as the limit of quantitation (LOQ).

Table 2 Analytical sensitivity of LOD and LOQ

Compound	LOD (μg/mL)	LOQ (μg/mL)
Gallic acid	0.15	0.5
Rutin	0.075	0.25
Quercetin	0.015	0.05
Kaempferol	0.015	0.05
Chlorogenic acid	0.1	0.5
Caffeic acid	0.05	0.1
Ferulic acid	0.1	0.5
p-Coumaric acid	0.1	0.5
Catechin	0.1	0.5
Ellagic acid	0.05	0.1

Table 3 The results of the analysis of bioactive compounds in the combined extract of mulberry leaves and butterfly pea flowers.

**Bioactive compounds**	**Concentration (Milligrams per 1 g of sample) (±SD)(n=3)**		
	**The extract of mulberry leaves**	**The extract of butterfly pea flowers**	**The combined extract of mulberry leaves and butterfly pea flowers (3:1)**
Gallic acid	-	0.376±0.017	0.143±0.003
Rutin	0.035±0.000	0.295±0.027	0.095±0.007
Quercetin	-	0.056±0.002	0.022±0.001
Kaempferol	-	0.049±0.001	0.041±0.000
Chlorogenic acid	0.063±0.001	-	0.052±0.000
p-Coumaric acid	-	-	-
Ellagic acid	0.011±0.001	0.056±0.003	0.030±0.001
Peonidin-3-O-glucoside	-	0.045±0.004	0.027±0.002

5. Conclusion

HPLC-DAD analysis of a 3:1 mixture of mulberry leaves extract and butterfly pea flowers extract, along with the combined extracts, showed the presence of ellagic acid, gallic acid, chlorogenic acid, rutin, quercetin, and kaempferol.
